# Risk and Protective Factors for Preterm Birth Among Racial, Ethnic, and Socioeconomic Groups in California

**DOI:** 10.1001/jamanetworkopen.2024.35887

**Published:** 2024-09-27

**Authors:** Laura L. Jelliffe-Pawlowski, Rebecca J. Baer, Scott Oltman, Safyer McKenzie-Sampson, Patience Afulani, Ribka Amsalu, April J. Bell, Bridgette Blebu, Kacie C.A. Blackman, Christina D. Chambers, Jean Costello, Jonathan Fuchs, Odessa Garay, Kayla L. Karvonen, Miriam Kuppermann, Audrey Lyndon, Charles E. McCulloch, Giannina Ong, Carolyn Ponting, Larry Rand, Elizabeth E. Rogers, Kelli K. Ryckman, Solaire Spellen, Akila Subramaniam, Louie Swander, Kelly D. Taylor, Schyneida Williams, Karen M. Tabb

**Affiliations:** 1 Department of Epidemiology and Biostatistics, University of California, San Francisco; 2 Department of Global Health Sciences, University of California, San Francisco; 3 California Preterm Birth Initiative, University of California, San Francisco; 4 Healthy Outcomes of Pregnancy for Everyone Research Consortium, University of California, San Francisco; 5 San Diego Study of Outcomes in Mothers and Infants, University of California San Diego, La Jolla; 6 EGG Healthy Pregnancy, San Francisco, California; 7 Department of Obstetrics, Gynecology, and Reproductive Sciences, University of California, San Francisco; 8 Department of Pediatrics, University of California San Diego, La Jolla; 9 Department of Pediatrics, Stanford University, Palo Alto, California; 10 Department of Family Community Medicine, University of California, San Francisco; 11 Lundquist Institute at Harbor-UCLA Medical Center, Torrance, California; 12 Department of Health Sciences, California State University, Northridge; 13 San Francisco Department of Public Health, San Francisco, California; 14 Department of Biology, San Francisco State University, San Francisco, California; 15 Department of Pediatrics, University of California, San Francisco; 16 Rory School of Nursing, New York University, New York, New York; 17 Department of Psychiatry, University of California, San Francisco; 18 Department of Epidemiology and Biostatistics, School of Public Health, Indiana University, Bloomington; 19 Departmment of Obstetrics and Gynecology, University of Alabama at Birmingham; 20 Department of Medicine, University of California, San Francisco; 21 Black Women’s Health and Livelihood Initiative, University of California, San Francisco; 22 School of Social Work, University of Illinois Urbana-Champaign, Urbana

## Abstract

**Importance:**

Preterm birth (PTB) (gestational age <37 weeks) is a major cause of infant mortality and morbidity in the US and is marked by racial and ethnic and socioeconomic inequities. Further research is needed to elucidate the association of risk and protective factors with trends in PTB rates and with related inequities.

**Objective:**

To describe the association of PTB rates with inequities as well as related risk and protective factors over the past decade in a US population–based cohort.

**Design, Setting, and Participants:**

This retrospective cohort study of singleton live births in California from January 1, 2011, to December 31, 2022, was conducted using vital statistics records and hospital records. The cohort included births with a gestational age of 22 to 44 weeks.

**Main Outcomes and Measures:**

Preterm birth rates by racial and ethnic group and by public and nonpublic insurance (considered as a proxy for socioeconomic status) were studied across years. Log-linear regression (relative risks with 95% CIs) was used to evaluate risk and protective factors within groups. Associations of PTB rates with risk and protective factors were assessed.

**Results:**

This study included 5 431 018 singleton live births to individuals who identified as American Indian or Alaska Native (0.3%), Asian (14.2%), Black (4.9%), Hispanic (47.8%), or White (27.0%). A total of 43.1% of births were to individuals with public health insurance. From 2011 to 2022, the overall PTB rate increased from 6.8% to 7.5% (change [SE], 10.6% [0.6%]; *z* score of 18.5; *P* < .001). Differences in PTB rates and associated changes were observed for racial and ethnic groups and insurance groups. For example, 2022 PTB rates ranged from 5.8% among White individuals with nonpublic insurance to 11.3% among Black individuals with public health insurance. From 2011 to 2022, PTB rates decreased from 9.1% to 8.8% (change [SE], −3.5% [4.2]; *z* score of −0.8; *P* = .42) among Black individuals with nonpublic insurance, whereas they increased from 6.4% to 9.5% (change [SE], 49.8% [16.0%]; *z* score of 3.1; *P* = .002) among American Indian or Alaska Native individuals with nonpublic insurance. Increases in some risk factors (eg, preexisting diabetes, sexually transmitted infections, mental health conditions) were observed in most groups, and decreases in some protective factors (eg, participation in the California Women, Infants, and Children program) (*P* for trend < .001 from 2011 to 2021) were observed mostly in low-income groups.

**Conclusions and Relevance:**

In this cohort study of singleton live births in California, PTB rates increased in many groups. Persistent racial and ethnic and socioeconomic inequities were also observed. Changes in risk and protective factors provided clues to patterns of PTB. These data point to an urgent need to address factors associated with PTB at both the individual and population levels.

## Key Points

**Question:**

What is the association of preterm birth (PTB) rates with related risk and protective factors as well as racial and ethnic and socioeconomic inequities?

**Findings:**

In this cohort study of 5 431 018 singleton births in California, PTB rates increased between 2011 and 2022 in most racial and ethnic groups and socioeconomic groups. Statistically significant increases in some risk factors (eg, preexisting diabetes, sexually transmitted infections, and mental health conditions) were observed across years and groups, as were decreases in some protective factors (eg, participation in supplemental nutrition programs) in low-income groups; racial and ethnic inequities in PTB rates persisted, as did socioeconomic inequities.

**Meaning:**

These findings suggest that there is an urgent need to address factors associated with PTB at both the individual and population levels.

## Teaser

This cohort study examines the association of preterm birth rates with related risk and protective factors as well as racial and ethnic and socioeconomic inequities in a California singleton birth cohort between 2011 and 2022.

## Introduction

Preterm birth (PTB) (gestational age <37 weeks) is a leading cause of infant mortality globally and is associated with both short- and long-term morbidity and mortality.^1^ In the US, PTB rates vary substantially, ranging from approximately 8% in Northeastern states such as Vermont and New Hampshire to more than 13% in Southern states such as Louisiana and Alabama.^2^ In February 2024, the Centers for Disease Control and Prevention (CDC) reported that PTB rates among singletons rose by 12% between 2014 and 2022.^3^

Inequities in PTB rates across racial and ethnic groups in the US have been well documented and continue to persist.^4^ For example, in 2022, the PTB rate was 14.6% among Black births compared with 10.1% among Hispanic births and 9.4% among White births.^4^ These inequities highlight the critical need for a comprehensive understanding of PTB rates and associated factors within different demographic groups. This knowledge is especially crucial for factors associated with increased risk of PTB for which effective treatments exist (eg, aspirin for preexisting hypertension, continuous glucose monitoring for diabetes, and counseling for mental health conditions) but are often underutilized and underprescribed, particularly in minoritized pregnant populations.^5,6,7,8,9,10^ Addressing these inequities through research and improved health care practices may potentially increase the utilization of these interventions and decrease the risk of PTB.

In December 2023, the US Centers for Medicare & Medicaid Services introduced the Transforming Maternal Health (TMaH) Model to enhance care for pregnant Medicaid recipients.^11^ The TMaH initiative is particularly important for addressing PTB-related inequities because it proposes a comprehensive approach to meet the multifaceted needs of vulnerable populations.^12^ This approach holds promise for addressing increasing PTB rates, given evidence that coordinated health and social services during pregnancy can lead to improved treatment uptake and lower PTB risk.^13,14,15^

In addition to informing clinical care practices and approaches, understanding group-specific patterns of and factors related to PTB is important for developing, planning, and monitoring public health programs, such as the TMaH Model. In this study, we examined patterns of PTB and related risk and protective factors from 2011 through 2022 within racial and ethnic groups in California by insurance status. Associations of PTB rates with risk and protective factors within these groups were also assessed.

## Methods

The protocol for this cohort study was approved by the Committee for the Protection of Human Subjects within the Health and Human Services Agency of California. In accordance with the Common Rule, informed consent was waived because publicly available data were used. The study adhered to the Strengthening the Reporting of Observational Studies in Epidemiology (STROBE) reporting guideline.

### Study Participants, Setting, and Sources

The study sample was drawn from a cohort of all singleton live births in California from January 1, 2011, through December, 31, 2022 (n = 5 638 355), and included those with gestational age from 22 to 44 weeks (n = 5 431 018 [96.3%]). Birth certificates from the California Department of Public Health Vital Records provided self-identified racial and ethnic grouping (considered as social factors in analyses). Racial and ethnic groupings included American Indian or Alaska Native, Asian, Black, Hispanic (Central or South American; Cuban; Mexican, Mexican American, or Chicano; Puerto Rican; or other Spanish or Hispanic ethnicity), Native Hawaiian or Other Pacific Islander, White, or other racial group (including Asian Indian, Filipino, ≥2 races, other specified racial group, refused to state, or unknown race or ethnicity). Additional information about racial and ethnic groups is included in eTable 1 in Supplement 1. Insurance group was used as a proxy for socioeconomic status (SES) and was categorized as public (Medi-Cal, California’s Medicaid program) or nonpublic (private insurance, self-pay, Indian Health Service, Civilian Health and Medical Program of Uniformed Service/Tricare, other payer, or unknown).

Preterm birth (gestational age 22 to <37 weeks) was based on best obstetric estimate. For 2011 to 2021 birth records, we were also able to link to hospital discharge, emergency department, and ambulatory surgery records from the California Department of Health Care Access and Information (2022 hospital records are not yet available). Linkage methods described elsewhere^16^ resulted in 89.1% of all singleton live births in California in 2011 to 2021 being included in analyses utilizing hospital-related records (n = 4 473 548 of 5 023 308). These records included maternal and infant characteristics, diagnoses, and procedures, coded according to *International Classification of Diseases, Ninth Revision* (*ICD-9*) and *Tenth Revision* (*ICD-10*) criteria.^17,18^ The *ICD-9* and *ICD-10* codes used are provided in eTable 1 in Supplement 1.

### Variables and Exposures

This study considered social determinants of health (SDOH) known to be associated with PTB risk or protection, including having public health insurance for prenatal care or childbirth, maternal place of birth, education level, number of prenatal care visits, smoking, drug or alcohol use, intimate partner violence (IPV), and housing insecurity.^19,20,21,22^ Also included were other physical and mental health factors consistently shown to be associated with PTB risk, available in the administrative database (many of which also have strong ties to SDOH).^23,24,25,26,27,28,29,30,31,32,33,34,35,36,37,38,39,40,41,42,43,44,45^ These factors included maternal age, prepregnancy body mass index (BMI [calculated as weight in kilograms divided by height in meters squared]), mental health condition, previous cesarean delivery or PTB, interpregnancy interval (IPI), diabetes, hypertension, preeclampsia, sickle cell anemia, non–sickle cell anemia, asthma, sleep disorders, autoimmune disorders, malignant neoplasms, dyslipidemia, infections, and urban vs rural Federal Information Processing Standard county code of residence.^46^ Enrollment in the California Women, Infants, and Children (WIC) program was also noted.^47^ eTable 1 in Supplement 1 details coding for composite variables.

###  Statistical Analysis

We used χ^2^ statistics to compare sample characteristics between individuals with and without PTB. Within-group changes in PTB rates between 2011 and 2022 were examined using percentage changes and associated SEs with evaluation of significance at 2-tailed *P* < .05 by *z* score. Log-linear regression with a Poisson distribution was used to assess PTB risk and protection within racial and ethnic groups and insurance groups using relative risks (RRs) and associated 95% CIs. To avoid incorrectly adjusting for variables important to the causal pathway (sometimes referred to as “overadjustment bias”),^48,49^ the study focused on well-established risk and protective factors and the presentation of RRs without multivariable adjustment.^19,20,21,22,23,24,25,26,27,28,29,30,31,32,33,34,35,36,37,38,39,40,41,42,43,44,45,48,49^ The Armitage-Cochrane test for trend assessed changes over time in PTB rates and in risk and protective factors by group, reported by 2-tailed *P* value. All analyses were conducted using SAS, version 9.4 (SAS Institute Inc).

## Results

### Patterns of PTB

This cohort study included 5 431 018 singleton live births among individuals who identified as American Indian or Alaska Native (0.3%), Asian (14.2%), Black (4.9%), Hispanic (47.8%), Native Hawaiian or Other Pacific Islander (0.4%), White (27.0%), or other racial or ethnic group (5.4%). Most births were to individuals who were aged 18 to 34 years (76.6%) and born in the US (63.3%). Approximately 39.4% of individuals were first-time parents, 43.1% used public insurance for childbirth, and 61.8% resided in urban areas (Table 1). From 2011 to 2022, the singleton PTB rate in California increased from 6.8% to 7.5% (change [SE], 10.6% [0.6%]; *z* score of 18.5; *P* < .001) (Table 2), with an observed *P* for trend < .001 across years (eTable 2 in Supplement 1). More than half of PTBs (54.7%) were spontaneous (eTable 3 in Supplement 1).

**Table 1.** Characteristics of California Singletons Born Between 2011 and 2022^a^

Characteristic	No. of births (%)		
	All (N = 5 431 018)	PTBs (n = 382 036)	Term births (n = 5 048 982)
Race and ethnicity			
American Indian or Alaska Native	17 773 (0.3)	1553 (0.4)	16220 (0.3)
Asian	768 372 (14.2)	51 776 (13.6)	716 596 (14.2)
Black	267 662 (4.9)	26 588 (7.0)	241 074 (4.8)
Hispanic^b^	2 595 477 (47.8)	192 677 (50.4)	2 402 800 (47.6)
Native Hawaiian or Other Pacific Islander	21 369 (0.4)	1831 (0.5)	19 539 (0.4)
White	1 466 030 (27.0)	84 619 (5.8)	1 381 411 (27.4)
Other^c^	294 335 (5.4)	22 992 (6.0)	271 343 (5.4)
Maternal age, y			
<18	72 825 (1.3)	6004 (1.6)	66 821 (1.3)
18-34	4 162 287 (76.6)	275 143 (72.0)	3 887 144 (77.0)
>34	1 195 538 (22.0)	100 827 (26.4)	1 094 711 (21.7)
Nulliparity	2 140 650 (39.4)	149 411 (39.1)	1 991 239 (39.4)
Public insurance	2 341 596 (43.1)	182 224 (47.7)	2 159 372 (42.8)
Education, y			
<12	777 299 (14.3)	63 456 (16.6)	713 843 (14.1)
12	1 314 195 (24.2)	97 848 (25.6)	1 216 347 (24.1)
>12	3 047 982 (56.1)	196 743 (51.5)	2 851 239 (56.5)
Place of birth for person giving birth			
US	3 439 077 (63.3)	245 464 (64.3)	3 193 613 (63.3)
Mexico	874 187 (16.1)	62 890 (16.5)	811 297 (16.1)
Other	1 991 941 (36.7)	136 572 (35.8)	1 855 369 (36.8)
FIPS county code of residence^46^			
1 (most urban)	3 357 627 (61.8)	324 290 (61.3)	3 123 337 (61.9)
2	696 745 (12.8)	49 242 (12.9)	647 503 (12.8)
3	1 034 207 (19.0)	74 989 (19.6)	959 218 (19.0)
4	224 522 (4.1)	15 510 (4.1)	209 012 (4.1)
5-6 (most rural)	94 073 (1.7)	6117 (1.6)	87 956 (1.7)

Abbreviations: FIPS, Federal Information Processing Standard; PTB, preterm birth (gestational age <37 completed weeks).

^a^ All row comparisons (yes vs no, by χ^2^ test) were significant at *P* < .001 except for FIPS code 2 (*P* = .25) and FIPS code 4 (*P* = .02).

^b^ Includes Central or South American; Cuban; Mexican, Mexican American, or Chicano; Puerto Rican; or other Spanish or Hispanic ethnicity. Additional information about racial and ethnic groups is included in eTable 1 in Supplement 1.

^c^ Includes Asian Indian, Filipino, 2 or more races, other specified racial group, refused to state, or unknown race or ethnicity.

**Table 2.** Change in PTB Rates by Insurance and Racial and Ethnic Groups, 2011 to 2022

Group	No. of PTBs/all births (%)		Change in PTB rate, 2011-2022		*P* value
	2011	2022	% (SE)	*z* Score	
Total cohort	33 069/484 612 (6.8)	30 760/407 710 (7.5)	10.6 (0.6)	18.5	<.001
Public insurance					
All births	16 549/228 535 (7.2)	13 825/164 401 (8.4)	16.1 (0.9)	17.8	<.001
Race and ethnicity^a^					
American Indian or Alaska Native	89/972 (9.2)	69/667 (10.3)	13.0 (15.8)	0.8	.42
Asian	974/13 370 (7.3)	723/8881 (8.1)	11.8 (3.8)	3.1	.002
Black	1467/14 561 (10.1)	1086/9648 (11.3)	11.7 (4.3)	2.7	.007
Hispanic	11 110/160 497 (6.9)	9275/113 179 (8.2)	18.4 (1.1)	17.1	<.001
Native Hawaiian or Other Pacific Islander	84/1064 (7.9)	63/679 (9.3)	17.5 (14.5)	1.2	.23
White	2261/31 351 (7.2)	1737/23 127 (7.5)	4.1 (2.4)	1.8	.07
Nonpublic insurance					
All births	16 520/256 077 (6.5)	16 935/243 309 (7.0)	7.9 (0.7)	11.3	<.001
Race and ethnicity					
American Indian or Alaska Native	47/740 (6.4)	53/557 (9.5)	49.8 (16.0)	3.1	.002
Asian	3145/46 439 (6.8)	3506/45 830 (7.7)	13.0 (1.8)	7.2	<.001
Black	1013/11 166 (9.1)	804/9187 (8.8)	−3.5 (4.2)	−0.8	.42
Hispanic	5934/83 050 (7.1)	6306/85 357 (7.4)	3.4 (1.3)	2.6	.01
Native Hawaiian or Other Pacific Islander	72/1017 (7.1)	68/865 (7.9)	11.0 (12.7)	0.9	.37
White	5471/101 418 (5.4)	4791/82 545 (5.8)	7.6 (1.1)	6.9	<.001

Abbreviation: PTB, preterm birth (gestational age <37 completed weeks).

^a^ Data not provided for other race or ethnicity due to insufficient power for specific group analyses.

**Figure 1.**
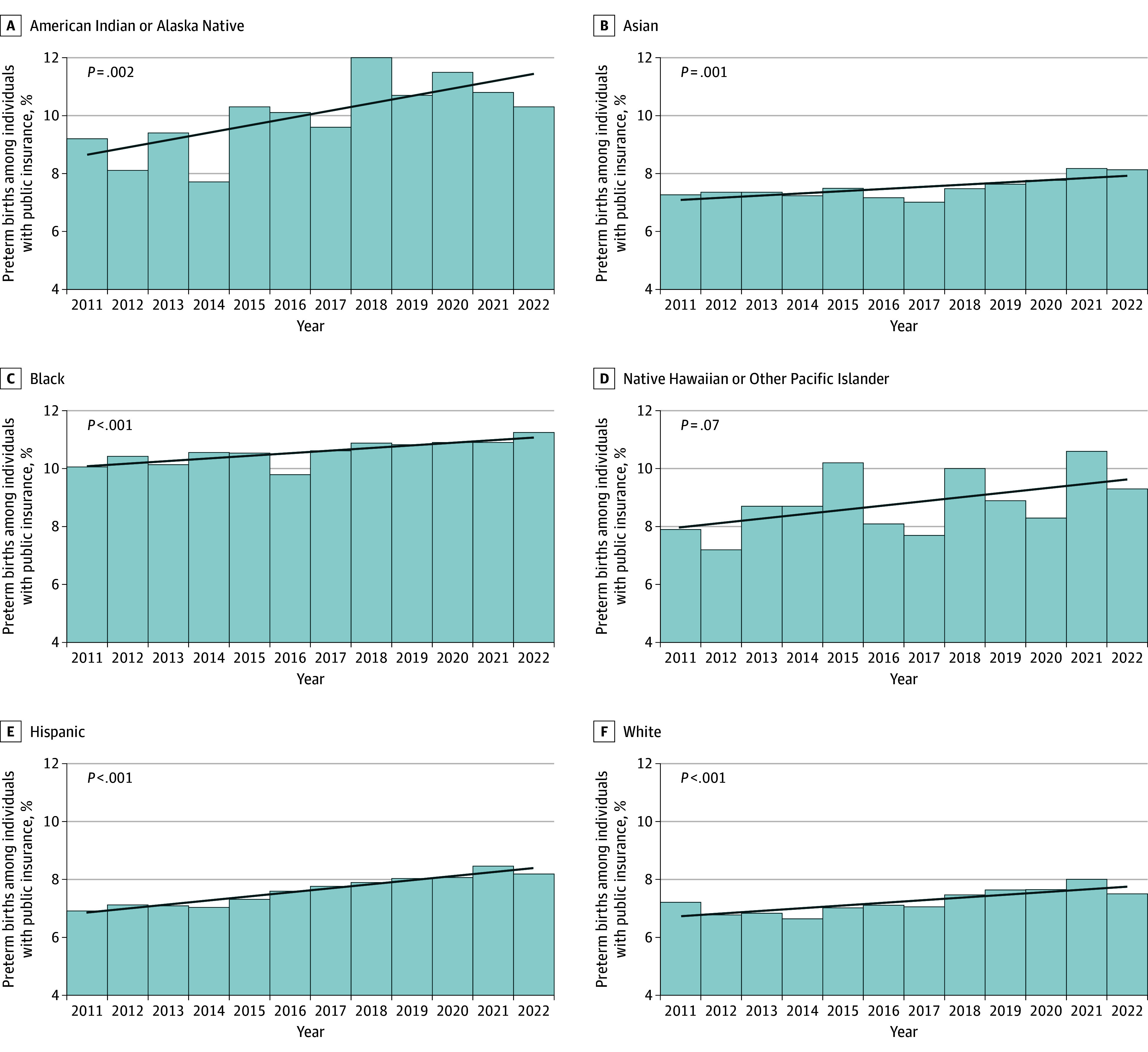
Preterm Birth (PTB) Rates Among California Singletons With Public Insurance by Racial and Ethnic Groups, 2011 to 2022 Gestational age of less than 37 completed weeks was considered preterm. *P* values were determined with the Armitage-Cochrane test for trend (2-tailed).

**Figure 2.**
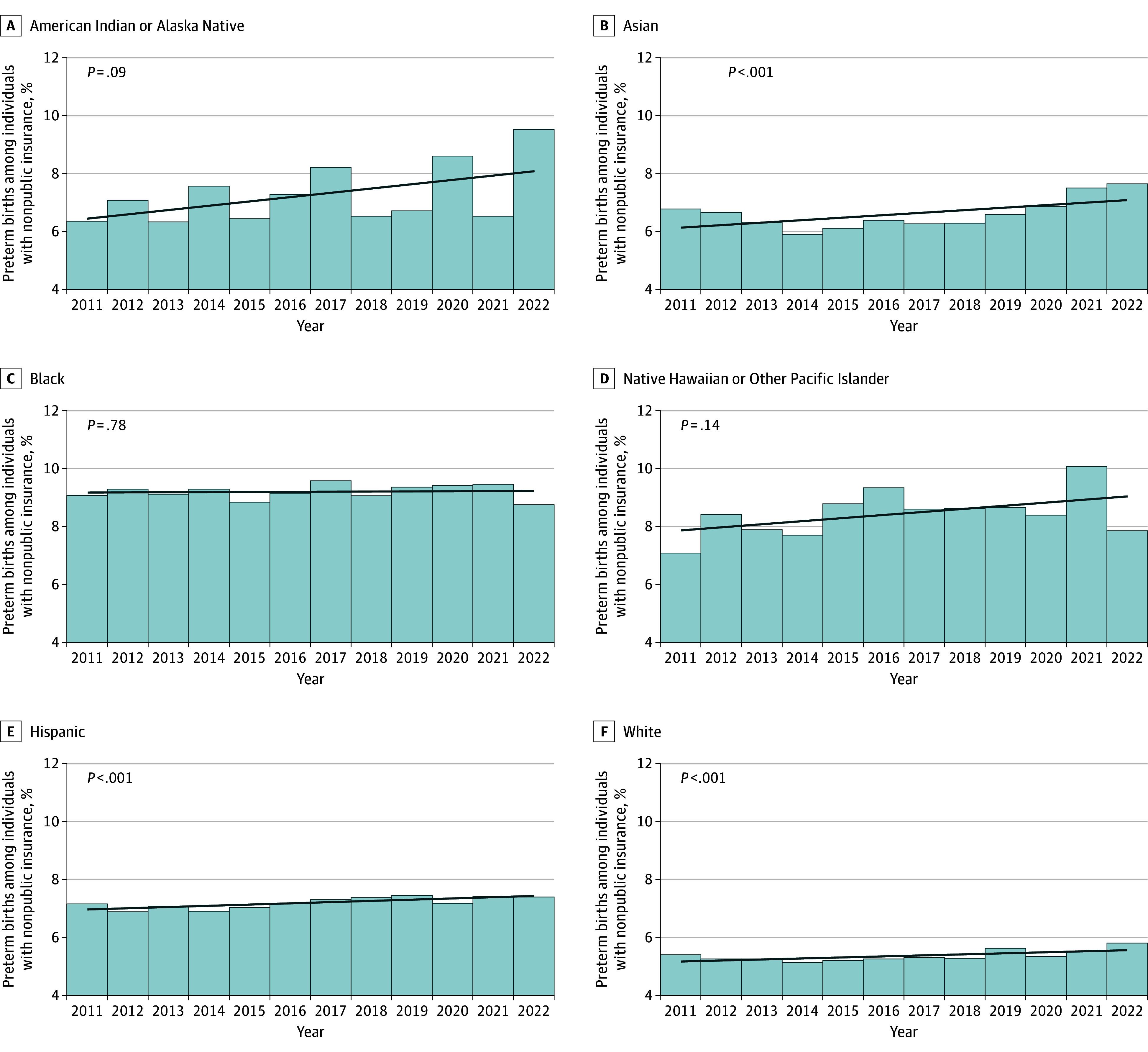
Preterm Birth (PTB) Rates Among California Singletons With Nonpublic Insurance by Racial and Ethnic Groups, 2011 to 2022 Gestational age of less than 37 completed weeks at birth was considered preterm. *P* values were determined with the Armitage-Cochrane test for trend (2-tailed).

The highest PTB rates were consistently observed among individuals who were American Indian or Alaska Native, Black, or Native Hawaiian or Other Pacific Islander and had public insurance (10.3%, 11.3%, and 9.3%, respectively, in 2022; Figure 1 and Table 2). The lowest PTB rates were most often observed in White individuals with nonpublic insurance (5.8% in 2022; Figure 2 and Table 2). From 2011 to 2022, PTB rates decreased from 9.1% to 8.8% (change [SE], −3.5% [4.2]; *z* score of −0.8; *P* = .42) among Black individuals with nonpublic insurance. However, significant increases in PTB rates were observed in most racial and ethnic groups by insurance type for the same period. For example, PTB rates increased from 7.1% in 2011 to 7.4% in 2022 among Hispanic individuals with nonpublic insurance (change [SE], 3.4% [1.3%]; *z* score of 2.6; *P* = 0.01 vs 18.4% [1.1%] for those with public insurance) and from 6.4% to 9.5% for American Indian or Alaska Native individuals with nonpublic insurance (change [SE], 49.8% [16.0%]; *z* score of 3.1; *P* = .002 vs 13.0% [15.8%] for those with public insurance). Across years, within-group associations between increasing PTB rates and public insurance were identified for all racial and ethnic groups except for Native Hawaiian or Other Pacific Islander individuals (Figure 1 and eTable 2 in Supplement 1). Associations between increasing PTB rates and nonpublic insurance were also observed for Asian, Hispanic, and White individuals (Figure 2 and eTable 2 in Supplement 1).

### Risk and Protective Factors

**Figure 3.**
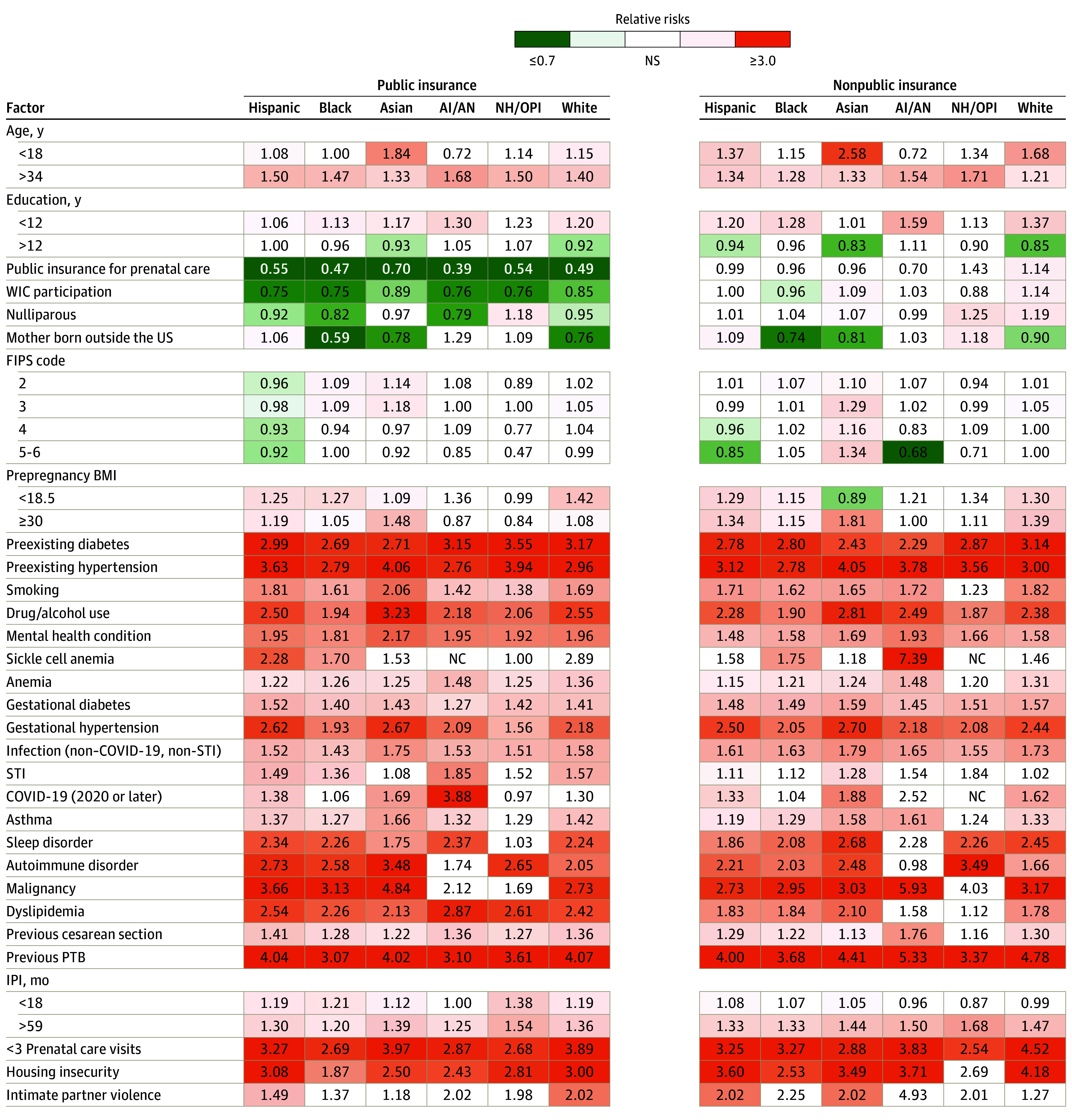
Risk and Protective Factors for Preterm Birth (PTB) by Insurance and Racial and Ethnic Groups Gestational age of less than 37 completed weeks was considered preterm. All comparisons were yes vs no except for age (18-34 years [referent]), education (12 years), FIPS county code of residence (1 [most urban]),^46^ BMI (18.5-29.9), and IPI (18-59 months). More complete coding for risk and protective factors is presented in eTable 1 in Supplement 1. AI/AN indicates American Indian or Alaska Native; BMI, body mass index (calculated as weight in kilograms divided by height in meters squared); FIPS, Federal Information Processing Standard (1 [most urban] to 6 [most rural]); IPI, interpregnancy interval; NC, not calculated (when n < 11 per state requirements [to protect individual-level privacy]); NH/OPI, Native Hawaiian or Other Pacific Islander; NS, nonsignificant; STI, sexually transmitted infection; WIC, California Women, Infants, and Children program.^47^

Associations (RRs ≥2) were observed between PTB rates and risk factors such as preexisting diabetes, preexisting hypertension, previous PTB, and fewer than 3 prenatal care visits (Figure 3 and eTable 4 in Supplement 1). In addition, associations (RRs >1) were observed between PTB rates and risk factors such as age older than 34 years, gestational diabetes or hypertension, mental health condition, substance use, non–sexually transmitted infection (STI) or COVID-19 infection, and IPI of more than 59 months. Conditions such as sickle cell anemia and autoimmune disorders were associated with higher risk in some groups, with housing insecurity notably associated with increasing PTB risk across all public insurance groups and in several nonpublic insurance groups, with RRs ranging from 1.87 (95% CI, 1.64-2.14; *P* < .001) for Black individuals with public insurance to 4.18 (95% CI, 3.35-5.22; *P* < .001) for White individuals with nonpublic insurance.

Public insurance coverage for prenatal care and delivery and WIC enrollment were consistently associated with decreasing risk for PTB across public insurance groups, with the most protection for having public insurance for prenatal care and delivery observed among American Indian or Alaska Native individuals (RR, 0.39 [95% CI, 0.32-0.48]; *P* < .001) and for being enrolled in WIC observed among individuals of other racial or ethnic groups (RR, 0.73 [95% CI, 0.70-0.77]; *P* < .001). Compared with Black individuals born in the US, those born outside of the US experienced lower PTB rates regardless of insurance type, with RRs ranging from 1.18 (95% CI, 1.01-1.39; *P* = .04) for the public insurance groups to 1.25 (95% CI, 1.08-1.46; *P* = .004) for the nonpublic insurance groups.

Large increases from 2011 to 2021 in several risk factors were observed across groups. For example, the rates of preexisting diabetes, STIs, and mental health conditions more than doubled (eTable 5 in Supplement 1). Other common risks with increases of greater than 20.0% across groups and years included maternal age greater than 34 years, prepregnancy BMI of 30 or greater, and gestational hypertension. Critically, housing insecurity rates more than doubled across years among most public insurance groups and also among American Indian or Alaska Native individuals and Black individuals with nonpublic insurance. In addition, IPV rates increased across years among Black individuals and Hispanic individuals in both the public and nonpublic insurance groups.

Reduced rates for several protective factors were also observed. Notably, WIC participation decreased substantially across most racial and ethnic groups with public insurance from 2011 to 2021 (eTable 5 in Supplement 1). A decreasing tendency for pregnant people with public insurance for their delivery to be enrolled in public insurance for their prenatal care was also observed.

## Discussion

In this study, a 10.6% increase in PTB rates among California singletons was observed from 2011 to 2022. The change over time differed by racial and ethnic groups and insurance groups, with the most pronounced increase (49.8%) observed among American Indian or Alaska Native individuals with nonpublic insurance. Individuals who were American Indian or Alaska Native, Black, or Native Hawaiian or Other Pacific Islander and had public health insurance consistently experienced the highest PTB rates (eg, 10.3%, 11.3%, and 9.3%, respectively, in 2022).

We also observed statistically significant shifts in PTB risk and protective factors across years and groups. Increases in risk factors such as preexisting diabetes, STIs, and mental health conditions were noted across most groups. Conversely, decreases in some protective factors (eg, WIC program participation) were observed mainly in the public insurance groups.

### PTB Rates and Trends

Our research supports recent CDC findings on increasing PTB rates,^3^ highlighting geographic and demographic patterns. The CDC reported a 12.0% increase in PTBs among singletons from 2014 to 2022, whereas we found a 10.6% increase from 2011 to 2022 in California specifically. We observed statistically significant variations in change over time by racial and ethnic group and by insurance group, including an 18.4% increase in the PTB rate among Hispanic individuals with public insurance compared with a 3.4% increase among those with nonpublic insurance. Our findings directly correspond to the TMaH initiative to target reductions in PTB rates through addressing SDOH.^11^

### Racial and Ethnic and Socioeconomic Inequities

Our study examined patterns of PTBs within racial and ethnic groups and SES-related groups, moving away from traditional comparisons that often use White individuals as a statistical referent.^50,51,52^ This method allows for an in-depth analysis of group-specific patterns, particularly associations with SDOH, and promotes discussion of how factors such as racism, discrimination, and resource availability affect observed patterns.^12,13^ This approach aims to better support pregnant individuals and new parents by acknowledging the role that social factors play in influencing risks and outcomes, thus helping to avoid mother-blame narratives.^53,54^

Our findings, consistent with other studies using a White referent,^50,51,52^ revealed statistically significant inequities in PTB rates among American Indian or Alaska Native, Black, and Native Hawaiian or Other Pacific Islander individuals. These inequities were present in public and nonpublic insurance groups, underscoring similar findings by others.^52^

### Risk and Protective Factors

To our knowledge, this study is one of the largest and most comprehensive efforts to date to examine associations between PTB patterns and risk and protective factors among racial and ethnic groups and socioeconomic groups in the US. This study extensively examined trends across groups and factors. Risk and protective factors studied were selected based on existing studies,^16,19,20,21,22,23,24,25,26,27,28,29,30,31,32,33,34,35,36,37,38,39,40,41,42,43,44,45,50,51,52^ and they aligned with known associations with PTB that were confirmed in this study.

Specific group risks were also confirmed in this study, including increased risk for PTB observed among Black populations with sickle cell anemia^55^ and among Black individuals with public insurance and housing insecurity.^37^ The protective role of WIC participation against PTB in public insurance groups was also validated.^56,57^

Importantly, this study also highlighted the protective association between place of birth (of the person giving birth) outside the US and PTB among Black individuals. This protection, attributed to less exposure to systemic racism, has been documented previously by our group and others.^31,36,58,59,60,61^ These findings underscore the importance of considering structural racism in PTB patterns. We also found that individuals who were Hispanic and born outside the US had a higher risk for PTB compared with their US-born peers, suggesting a possible association between discrimination related to immigration and PTB, as has been reported by other investigators.^62,63,64^

### Trends in Risk and Protective Factors

Our findings of increasing rates of known PTB risks, such as preexisting diabetes and gestational hypertension, and of mental health conditions across SES groups also align with other studies,^65,66,67^ as do our results with respect to increasing rates of maternal obesity and STIs during pregnancy.^68,69^ Importantly, our findings showing an increased number of individuals reporting housing insecurity while pregnant, particularly those with public health insurance, align with those of other investigators who have reported this increase,^70,71^ as do our findings of increasing IPV rates in some groups.^70^ Our observation of decreasing WIC participation rates in low-income populations has also been reported by others in differing locations.^72^

### Applying the Data

The findings of this study highlight the crucial need for personalized, multisector coordinated care during pregnancy to address health and social risks for PTB. Although this need spans the entire population, the recently introduced TMaH Model^11^ provides a blueprint for such coordination for pregnant individuals with Medicaid or Medi-Cal insurance.

Critically, the study data underscore the need for better utilization of interventions shown to reduce the risk of PTB and related adverse outcomes (eg, preeclampsia and intrauterine growth restriction) in individuals with specific risk factors. This includes improved use of low-dose aspirin among pregnant individuals with increased risk due to its established efficacy in decreasing risk for PTB and related conditions such as preeclampsia.^5,73^ For example, data show that fewer than 1 in 3 pregnant individuals who should be recommended low-dose aspirin based on the presence of 1 or more major risk factors (eg, personal history of preeclampsia, chronic hypertension, or type 1 or 2 diabetes) or 2 or more moderate risk factors (eg, nulliparity, BMI ≥30, Black race, or age ≥35 years) receive a prescription.^5,73^ Other underutilized interventions include continuous glucose monitoring for type 1 diabetes,^6,74^ screening and treatment for STIs,^10^ and support for mental health conditions.^8,75^ Additional underutilized interventions during pregnancy relate to smoking cessation^7^ and treatment of urinary tract infections,^76^ asthma,^77^ autoimmune disorders,^78^ and cancer.^79^ Given the observed increases in housing insecurity among the public insurance group and IPV among Black or Hispanic individuals in this study, it is also essential to improve public health efforts connecting pregnant people with services focused on these factors.^70,71,80,81,82^ Increasing the prescription and uptake of proven interventions is particularly important in racial minority populations, in which underutilization and underprescription has been shown to be especially profound.^5,6,7,8,9,10,74,75,76,77,78,79,80,81,82^

Our findings suggest that consideration of the presence of a multitude of risk factors for PTB may be beneficial for initiatives such as the TMaH Model^11^ in helping health care clinicians, coordinators, and patients address personal risk and enhance their overall awareness about PTB (including early warning signs and interventions that may be helpful to infants in the case of premature labor).^83,84,85,86^ Importantly, these efforts would benefit from ensuring a focus on patient preferences in risk communication.^87,88,89,90,91,92,93,94^

The decreasing trend in WIC enrollment in California over time has also been reported nationally^95^ and is particularly concerning, given the observed association between WIC participation and lower PTB rates demonstrated in this and previous studies.^56,57^ Factors contributing to decreased participation have been identified as including language barriers, education, and stigma.^95,96,97^ Strategies shown to lead to improvement in enrollment include outreach to eligible pregnant people and their clinicians and antiracism training for staff.^97,98^ These findings underscore the potential for interventions to increase WIC enrollment and, in turn, potentially increase protection against PTB in some pregnancies.

### Strengths and Limitations

Strengths of this study include its examination of within-group patterns over time in a large population-based dataset with extensive demographic, SDOH, and clinical factor data present. Despite these strengths, this study has some limitations, including a lack of data on the interventions used and limited numbers for some racial and ethnic groups (eg, Filipino and multiple races). Future research should expand to a national sample to allow for a more detailed analysis of these groups and also to explore patterns in other geographies and public health systems. Importantly, although we focused on bivariate associations to avoid overadjustment bias,^48,49^ further analyses with adjusted factors may prove helpful in efforts aimed at elucidating aggregate risk.^44,45^ Examination by spontaneous and medically indicated PTB subgroups may also be important in future investigations because trends and associated factors may differ within these groups.^99,100^ Also, the current dataset likely includes individuals who gave birth multiple times. No statewide identifier for mothers across pregnancies and years exists, making this examination in California challenging. Follow-up investigation will be important in this respect.

## Conclusions

This cohort study revealed trends and factors that likely contribute to racial and ethnic and socioeconomic inequities in PTB rates among California singleton births. In particular, this study highlighted increasing PTB rates and ongoing inequities, particularly among American Indian or Alaska Native, Black, Native Hawaiian or Other Pacific Islander, and low-income groups. These results underscore the need to enhance access to personalized pregnancy care and to promote treatments for conditions such as preexisting and gestational diabetes and hypertension, STIs, and mental health conditions. Additionally, these data suggest that encouraging early prenatal care and participation in support programs, such as WIC, may help mitigate risk for PTB and other adverse pregnancy outcomes. Critically, these data provide actionable information that can be used to inform efforts aimed at improving reproductive health equity through clinical and other public health efforts to address individual and population-level risks and to promote related protective factors.

## Notes

Supplement 1. **eTable 1.** Coding Schema for Summary Variables **eTable 2.** Rates and Trends in Preterm Birth: California Singleton Births by Insurance and Racial and Ethnic Groups From 2011 to 2022 **eTable 3.** Preterm Birth (PTB) by Timing and Subtype: California Singleton Births in 2011 to 2022 With Recorded Gestational Weeks on Birth Certificates From 22 to 44 Weeks **eTable 4.** Risk and Protection for Preterm Birth (PTB) by Relative Risks (RRs) and 95% CIs: California Singleton Births by Insurance and Racial and Ethnic Groups From 2011 to 2022 **eTable 5.** Pattern Over Time of Risk and Protective Factors for Preterm Birth (PTB) (by Percent) With Test for Trend: California Singleton Births by Insurance and Racial and Ethnic Groups From 2011 to 2022 (Presented for Factors Where Significant in eTable 4)
eReferences

Supplement 2.
Data Sharing Statement
