# Supplementary material for: Risk and Protective Factors for Preterm Birth Among Racial, Ethnic, and Socioeconomic Groups in California
Source: JAMA Netw Open. 2024 Sep 27;7(9):e2435887. doi: 10.1001/jamanetworkopen.2024.35887 (PMC11437386; doi:10.1001/jamanetworkopen.2024.35887)
Supplement: Supplement 1. — eTable 1. Coding Schema for Summary Variables eTable 2. Rates and Trends in Preterm Birth: California Singleton Births by Insurance and Racial and Ethnic Groups From 2011 to 2022 eTable 3. Preterm Birth (PTB) by Timing and Subtype: California Singleton Births in 2011 to 2022 With Recorded Gestational Weeks on Birth Certificates From 22 to 44 Weeks eTable 4. Risk and Protection for Preterm Birth (PTB) by Relative Risks (RRs) and 95% CIs: California Singleton Births by Insurance and Racial and Ethnic Groups From 2011 to 2022 eTable 5. Pattern Over Time of Risk and Protective Factors for Preterm Birth (PTB) (by Percent) With Test for Trend: California Singleton Births by Insurance and Racial and Ethnic Groups From 2011 to 2022 (Presented for Factors Where Significant in eTable 4) eReferences [file jamanetwopen-e2435887-s001.pdf]

Supplementary Online Content

Jelliffe-Pawlowski LL, Baer RJ, Oltman S, et al. Risk and protective factors for preterm birth among racial, ethnic, and socioeconomic groups in California. *JAMA Netw Open*. 2024;7(10):e2435887. doi:10.1001/jamanetworkopen.2024.35887

eTable 1. Coding Schema for Summary Variables

eTable 2. Rates and Trends in Preterm Birth: California Singleton Births by Insurance and Racial and Ethnic Groups From 2011 to 2022

eTable 3. Preterm Birth (PTB) by Timing and Subtype: California Singleton Births in 2011 to 2022 With Recorded Gestational Weeks on Birth Certificates From 22 to 44 Weeks

eTable 4. Risk and Protection for Preterm Birth (PTB) by Relative Risks (RRs) and 95% CIs: California Singleton Births by Insurance and Racial and Ethnic Groups From 2011 to 2022

eTable 5. Pattern Over Time of Risk and Protective Factors for Preterm Birth (PTB) (by Percent) With Test for Trend: California Singleton Births by Insurance and Racial and Ethnic Groups From 2011 to 2022 (Presented for Factors Where Significant in eTable 4)

eReferences

This supplementary material has been provided by the authors to give readers additional information about their work.

© 2024 Jelliffe-Pawlowski LL et al. *JAMA Network Open.*

eTable 1. Coding Schema for Summary Variables[a]

Page 1 of 2

	**Maternal ICD-9**[1]	**Maternal ICD-10**[2] **/ Vital Statistics Code**	**Infant ICD-9**[1]	**Infant ICD-10**[2]
**Racial/Ethnicity Group** (additional information not in methods section)				
Asian		/'Asian-unspecified', 'Asian-specified', 'Asian-Chinese', 'Asian-Japanese', 'Asian-Korean', 'Asian-Vietnamese', 'Asian-Cambodian', 'Asian-Thai', 'Asian-Lao', 'Asian-Hmong'		
Hawaiian/Pacific Islander		/'Hawaiian', 'Guamanian', 'Samoan', 'Other Pacific Islander'		
Other		/'Two or more races', 'Indian (Asian)', 'Filipino', 'Other-specified', 'Refused to state', 'Unknown'		
**Preterm Birth (PTB) Subtypes**				
Spontaneous PTB with Premature Rupture of the Membranes (PPROM)[b]	658.1	O42	761.1	P01.1
Spontaneous PTB with Intact Membranes[c]	644	O60		
Provider Initiated PTB[d]	669.7, procedure code 73.0, 73.1, 73.4, 74	O82, procedure code 10907ZC, 3E033VJ, 3E0P7GC, 10D00Z0, 10D00Z1, 10D00Z2	763.4	P03.4
**Conditions/ Diagnoses Present Before and During Pregnancy**				
Preexisting Diabetes	648.0, 249, 250	O24.0, O24.1, O24.2, O24.3, E10, E11, E12, E13, E14	P70.1	
Preexisting Hypertension	642.0, 642.1, 642.2	O10		
Sickle Cell Anemia	282.6, 282.41, 282.42	D57.0, D57.1, D57.2		
**Diagnoses/Conditions During Pregnancy**				
Gestational Diabetes	648.8	O24.4	775.0	P70.0
Gestational Hypertension	642.3	O13	760.0	P00.0
Sexually Transmitted Infection	647.0, 647.1, 647.2	O98.1, O98.2, O98.3		
COVID-19[e]		U07.1		
Other Infection	656.5, 656.6, 647.3, 647.4, 647.5, 647.6, 647.7, 647.8, 647.9	O23.0, O23.1, O23.2, O23.3, O23.4, O98.0, O98.4, O98.5, O98.6, O98.7, O98.8, O98.9		
Asthma[f]	493	J45		
Sleep Disorder[f]	780.52, 307.41, 327.1, 780.57, 780.53, 780.51, 327.2	F51.0, G47.0, G47.3		
Autoimmune Disorder[f]	289.81, 646.8, 696.0, 710.0, 714.0, 714.3, 720.0	M05, M07, M08, M32, M45, M09, L40.5, D68.5, D68.6		
Malignancy[f]	140 – 209.9	Any first letter 'C'		
Dyslipidemia[f]	272.0, 272.1, 272.2, 272.3, 272.4, 272.5	E78		

© 2024 Jelliffe-Pawlowski LL et al. *JAMA Network Open.*

eTable 1. (continued)

Page 2 of 2

	**Maternal ICD-9**[1]	**Maternal ICD-10**[2] **/ Vital Statistics Code**	**Infant ICD-9**[1]	**Infant ICD-10**[2]
**Diagnoses/Conditions During Pregnancy** (continued)				
Mental Health Condition[f]	648.4	O99.3		
Anemia[f]	648.2	O99.0		
Housing Insecurity[f]	V60.0, V60.1	Z59.0, Z59.1		
Interpersonal Violence[f]	995.80, 995.81, 995.82, 995.83, 995.84, 995.85	T74.01XA, T74.11XA, T74.21XA, T74.31XA, T76.01XA, T76.11XA, T76.21XA, T76.31XA, O9A311, O9A312, O9A313, O9A319, O9A32, O9A33, O9A411, O9A412, O9A413, O9A419, O9A42, O9A43, O9A511, O9A512, O9A513, O9A519, O9A52, O9A53		

Abbreviations: PPROM, preterm premature rupture of the membranes; PTB, preterm birth (gestational weeks < 37 completed weeks)
[a]Described for variables where less detail is provided in methods section
[b]< 37 completed weeks gestation, indication of premature rupture of the membranes
[c]< 37 completed weeks gestation, indication of tocolytic medication (on birth certificate) or preterm labor (in hospital discharge records), no indication of PPROM
[d]< 37 completed weeks gestation, no indication of preterm labor or PPROM, with an indication of induction, augmented labor, artificial rupture of membranes, or cesarean delivery on birth certificate records or in hospital discharge records
[e]Coded for 2020 births forward
[f]Coded as present during pregnancy but may also have been present prior to pregnancy

© 2024 Jelliffe-Pawlowski LL et al. *JAMA Network Open.*

eTable 2. Rates and Trends in Preterm Birth: California Singleton Births by Insurance and Racial and Ethnic Groups From 2011 to 2022

Page 1 of 2

		2011	2012	2013	2014	2015	2016	2017	2018	2019	2020	2021	2022	*P* (trend)
Sample														
	All births	484,612	485,509	476,412	484,303	474,192	471,652	455,371	441,436	433,217	408,181	408,423	407,710	
	PTB	33,069	33,075	32,172	32,061	32,252	32,738	32,084	31,540	31,719	29,611	30,955	30,760	
	**PTB %**	**6.8%**	**6.8%**	**6.8%**	**6.6%**	**6.8%**	**6.9%**	**7.0%**	**7.1%**	**7.3%**	**7.3%**	**7.6%**	**7.5%**	**< .001**
Public Insurance														
All	Births	228,535	226,018	216,675	213,352	208,170	204,839	196,226	184,616	174,114	163,146	161,504	164,401	
	PTB	16,549	16,672	15,900	15,530	15,693	15,780	15,447	14,984	14,360	13,500	13,984	13,825	
	**PTB %**	**7.2%**	**7.4%**	**7.3%**	**7.3%**	**7.5%**	**7.7%**	**7.9%**	**8.1%**	**8.2%**	**8.3%**	**8.7%**	**8.4%**	**< .001**
Hispanic	All births	160,497	156,506	150,873	147,619	144,165	139,732	133,523	125,022	115,893	108,175	107,963	113,179	
	PTB	11,110	11,155	10,695	10,394	10,560	10,626	10,379	9,871	9,322	8,734	9,143	9,275	
	**PTB %**	**6.9%**	**7.1%**	**7.1%**	**7.0%**	**7.3%**	**7.6%**	**7.8%**	**7.9%**	**8.0%**	**8.1%**	**8.5%**	**8.2%**	**<.001**
Non-Hispanic														
Black	All births	14,561	14,469	13,937	13,563	12,628	12,475	11,964	11,400	11,089	10,378	10,226	9,648	
	PTB	1,467	1,509	1,414	1,434	1,332	1,222	1,271	1,241	1,201	1,132	1,116	1,086	
	**PTB %**	**10.1%**	**10.4%**	**10.1%**	**10.6%**	**10.5%**	**9.8%**	**10.6%**	**10.9%**	**10.8%**	**10.9%**	**10.9%**	**11.3%**	**<.001**
Asian	All births	13,370	14,606	13,356	13,661	13,169	13,656	13,289	12,097	11,374	9,734	9,182	8,881	
	PTB	974	1,077	983	989	988	981	933	906	869	757	752	723	
	**PTB %**	**7.3%**	**7.4%**	**7.4%**	**7.2%**	**7.5%**	**7.2%**	**7.0%**	**7.5%**	**7.6%**	**7.8%**	**8.2%**	**8.1%**	**0.001**
American Indian/	All births	972	983	946	944	881	786	792	811	773	712	695	667	
Alaska Native	PTB	89	80	89	73	91	79	76	104	83	82	75	69	
	**PTB %**	**9.2%**	**8.1%**	**9.4%**	**7.7%**	**10.3%**	**10.1%**	**9.6%**	**12.8%**	**10.7%**	**11.5%**	**10.8%**	**10.3%**	**0.002**
Hawaiian/	All births	1,064	985	958	952	933	889	859	837	745	703	663	679	
Pacific Islander	PTB	84	71	83	83	95	72	66	84	66	58	70	63	
	**PTB %**	**7.9%**	**7.2%**	**8.7%**	**8.7%**	**10.2%**	**8.1%**	**7.7%**	**10.0%**	**8.9%**	**8.3%**	**10.6%**	**9.3%**	**0.067**
Other	All births	6,620	7,700	6,199	6,774	6,930	8,157	7,362	8,086	8,898	8,757	8,462	8,220	
	PTB	564	693	558	576	588	729	715	808	882	849	886	872	
	**PTB %**	**8.5%**	**9.0%**	**9.0%**	**8.5%**	**8.5%**	**8.9%**	**9.7%**	**10.0%**	**9.9%**	**9.7%**	**10.5%**	**10.6%**	**<.001**
White	All births	31,351	30,769	30,406	29,839	29,464	29,144	28,437	26,363	25,342	24,687	24,131	23,127	
	PTB	2,261	2,087	2,078	1,981	2,069	2,071	2,007	1,970	1,937	1,888	1,951	1,737	
	**PTB %**	**7.2%**	**6.8%**	**6.8%**	**6.6%**	**7.0%**	**7.1%**	**7.1%**	**7.5%**	**7.6%**	**7.6%**	**8.1%**	**7.5%**	**<.001**

© 2024 Jelliffe-Pawlowski LL et al. *JAMA Network Open.*

eTable 2. (continued)

Page 2 of 2

		2011	2012	2013	2014	2015	2016	2017	2018	2019	2020	2021	2022	*P* (trend)
Non-Public Insurance														
All	Births	256,077	259,491	259,737	270,951	266,022	266,813	259,145	256,820	259,103	245,035	246,919	243,309	
	PTB	16,520	16,403	16,272	16,531	16,559	16,958	16,637	16,556	17,359	16,111	16,971	16,935	
	**PTB %**	**6.5%**	**6.3%**	**6.3%**	**6.1%**	**6.2%**	**6.4%**	**6.4%**	**6.4%**	**6.7%**	**6.6%**	**6.9%**	**7.0%**	**<.001**
Hispanic	All births	83,050	81,963	81,170	83,383	83,693	82,959	80,700	81,581	83,518	81,644	83,212	85,357	
	PTB	5,934	5,641	5,735	5,753	5,935	5,955	5,891	6,016	6,219	5,862	6,175	6,306	
	**PTB %**	**7.1%**	**6.9%**	**7.1%**	**6.9%**	**7.1%**	**7.2%**	**7.3%**	**7.4%**	**7.4%**	**7.2%**	**7.4%**	**7.4%**	**<.001**
Non-Hispanic														
Black	All births	11,166	10,624	10,534	10,327	10,074	10,034	10,006	9,859	9,995	9,679	9,839	9,187	
	PTB	1,013	987	961	959	891	918	959	893	936	911	931	804	
	**PTB %**	**9.1%**	**9.3%**	**9.1%**	**9.3%**	**8.8%**	**9.1%**	**9.6%**	**9.1%**	**9.4%**	**9.4%**	**9.5%**	**8.8%**	**0.777**
Asian	All births	46,439	51,454	52,274	58,550	55,196	57,355	55,991	54,019	53,557	46,672	44,660	45,830	
	PTB	3,145	3,425	3,303	3,457	3,370	3,664	3,507	3,390	3,535	3,204	3,348	3,506	
	**PTB %**	**6.8%**	**6.7%**	**6.3%**	**5.9%**	**6.1%**	**6.4%**	**6.3%**	**6.3%**	**6.6%**	**6.9%**	**7.5%**	**7.7%**	**<.001**
American Indian/	All births	740	693	743	727	683	659	621	629	641	581	537	557	
Alaska Native	PTB	47	49	47	55	44	48	51	41	43	50	35	53	
	**PTB %**	**6.4%**	**7.1%**	**6.3%**	**7.6%**	**6.4%**	**7.3%**	**8.2%**	**6.5%**	**6.7%**	**8.6%**	**6.5%**	**9.5%**	**0.086**
Hawaiian/	All births	1,017	998	976	986	990	889	895	834	901	857	894	865	
Pacific Islander	PTB	72	84	77	76	87	83	77	72	78	72	90	68	
	**PTB %**	**7.1%**	**8.4%**	**7.9%**	**7.7%**	**8.8%**	**9.3%**	**8.6%**	**8.6%**	**8.7%**	**8.4%**	**10.1%**	**7.9%**	**0.141**
Other	All births	12,247	13,867	13,168	14,855	15,231	17,539	17,470	18,607	20,097	19,528	20,611	18,968	
	PTB	838	969	857	994	1,032	1,181	1,204	1,332	1,481	1,418	1,589	1,407	
	**PTB %**	**6.8%**	**7.0%**	**6.5%**	**6.7%**	**6.8%**	**6.7%**	**6.9%**	**7.2%**	**7.4%**	**7.3%**	**7.7%**	**7.4%**	**0.214**
White	All births	101,418	99,892	100,872	102,123	100,173	97,378	93,462	91,291	90,394	86,074	87,166	82,545	
	PTB	5,471	5,248	5,292	5,237	5,200	5,109	4,948	4,812	5,077	4,594	4,803	4,791	
	**PTB %**	**5.4%**	**5.3%**	**5.2%**	**5.1%**	**5.2%**	**5.2%**	**5.3%**	**5.3%**	**5.6%**	**5.3%**	**5.5%**	**5.8%**	**<.001**

Abbreviations: PTB, preterm birth (gestational weeks < 37 completed weeks)
Racial/ethnicity groups other than 'Hispanic" are non-Hispanic, 'Other' race/ethnicity = 'Indian (Asian)', 'Filipino', 'two or more races', 'other-specified', 'refused to state', and 'unknown'. Additional information about race/ethnicity groupings is included in eTable 1.

© 2024 Jelliffe-Pawlowski LL et al. *JAMA Network Open.*

eTable 3. Preterm Birth (PTB) by Timing and Subtype[a]: California Singleton Births in 2011 to 2022 With Recorded Gestational Weeks on Birth Certificates From 22 to 44 Weeks

	n =	%
**%, Full Sample**		
All singleton births	5,431,018	100.0
Any PTB (< 37 weeks)	382,036	7.0
Early PTB (< 32 weeks)	53,649	1.0
Late PTB (32-36 weeks)	328,387	6.0
**%, Any PTB (< 37 weeks)**	382,036	100.0
Spontaneous	208,955	54.7
PPROM	84,467	22.1
Spontaneous labor with intact membranes	124,488	32.6
Provider Initiated	76,875	20.1
Unknown	96,206	25.2
**%, Early PTB (< 37 weeks)**	53,649	100.0
Spontaneous	30,360	56.6
PPROM	13,101	24.4
Spontaneous labor with intact membranes	17,259	32.2
Provider Initiated	9,512	17.7
Unknown	13,777	25.7
**%, Late PTB (32-36 weeks)**	328,387	100.0
Spontaneous	178,595	54.4
PPROM	71,366	21.7
Spontaneous labor with intact membranes	107,229	32.7
Provider Initiated	67,363	20.5
Unknown	82,429	25.1

Abbreviations: PTB, preterm birth (gestational weeks < 37 completed weeks)
[a]See coding as described in eTable 1 (full coding of subtypes available for births in 2011-2021, coding of subtypes for 2022 births based solely on birth certificate records)

© 2024 Jelliffe-Pawlowski LL et al. *JAMA Network Open.*

eTable 4. Risk and Protection for Preterm Birth (PTB) by Relative Risks (RRs) and 95% CIs[a,b]: California Singleton Births by Insurance and Racial and Ethnic Groups From 2011 to 2022

Page 1 of 7

	American Indian/Alaska Native							
	Public Insurance				Non-Public Insurance			
**Risk/Protective Factors**	**PTB %**	**Term %**	**RR**	**95% CI**	**PTB %**	**Term %**	**RR**	**95% CI**
Age < 18 years	1.7	2.6	0.72	0.42 - 1.25	0.8	1.2	0.72	0.23 - 2.25
Age > 34 years	18.7	11.3	1.68	1.40 - 2.01	28.1	19.5	1.54	1.24 - 1.92
< 12 years education	27.0	22.3	1.30	1.09 - 1.54	11.5	7.9	1.59	1.12 - 2.24
> 12 years education	31.2	32.6	1.05	0.89 - 1.24	57.6	57.8	1.11	0.89 - 1.40
Public insurance for prenatal care	87.6	95.5	0.39	0.32 - 0.48	1.5	2.2	0.70	0.31 - 1.58
WIC participation	65.4	72.0	0.76	0.65 - 0.88	30.6	30.0	1.03	0.83 - 1.27
Nulliparous	23.1	27.9	0.79	0.67 - 0.94	34.8	35.1	0.99	0.81 - 1.22
Mom born outside the US	1.5	1.2	1.29	0.73 - 2.28	3.3	3.2	1.03	0.59 - 1.80
FIPS 2	8.9	8.0	1.08	0.83 - 1.42	12.0	10.4	1.07	0.78 - 1.47
FIPS 3	23.8	23.3	1.00	0.83 - 1.21	18.6	16.9	1.02	0.78 - 1.33
FIPS 4	14.5	12.9	1.09	0.87 - 1.37	7.3	8.3	0.83	0.56 - 1.23
FIPS 5-6	22.0	25.9	0.85	0.70 - 1.03	13.5	19.0	0.68	0.50 - 0.92
Pre-pregnancy BMI < 18.5 kg/m2	3.6	2.4	1.36	0.92 - 2.00	2.8	2.3	1.21	0.65 - 2.24
Pre-pregnancy BMI ≥ 30 kg/m2	34.1	37.4	0.87	0.73 - 1.03	34.1	34.9	1.00	0.79 - 1.27
Preexisting Diabetes	6.8	1.8	3.15	2.38 - 4.16	4.3	1.7	2.29	1.41 - 3.73
Preexisting Hypertension	10.1	3.3	2.76	2.19 - 3.48	11.5	2.7	3.78	2.78 - 5.14
Smoking	23.3	17.0	1.42	1.21 - 1.68	10.8	6.3	1.72	1.25 - 2.36
Drug/Alcohol Use	23.8	11.4	2.18	1.85 - 2.57	12.8	5.0	2.49	1.86 - 3.34
Mental Health Condition	32.4	18.3	1.95	1.68 - 2.27	21.3	11.7	1.93	1.52 - 2.45
Sickle Cell Anemia	0.0	0.1	n/c	n/c	0.3	0.0	7.39	1.04 - 52.51
Anemia	19.0	13.1	1.48	1.24 - 1.77	18.3	12.8	1.48	1.15 - 1.90
Gestational Diabetes	12.4	9.8	1.27	1.02 - 1.57	15.8	11.2	1.45	1.11 - 1.89
Gestational Hypertension	16.6	7.9	2.09	1.73 - 2.52	18.1	8.5	2.18	1.69 - 2.81
Infection (non-COVID-19, non-STI)	23.2	15.8	1.53	1.30 - 1.81	15.3	9.4	1.65	1.26 - 2.17
Sexually Transmitted Infection	3.8	1.9	1.85	1.28 - 2.55	2.0	1.3	1.54	0.77 - 3.11
COVID-19 (2020+ only)	8.5	1.7	3.88	1.55 - 9.69	4.8	1.7	2.52	0.61 - 10.44
Asthma	14.5	11.1	1.32	1.08 - 1.61	12.3	7.7	1.61	1.20 - 2.18
Sleep Disorder	1.0	0.4	2.37	1.18 - 4.75	1.0	0.4	2.28	0.85 - 6.11
Autoimmune Disorder	1.2	0.6	1.74	0.90 - 3.36	0.5	0.5	0.98	0.25 - 3.95
Malignancy	0.3	0.1	2.12	0.53 - 8.49	0.5	0.1	5.93	1.48 - 23.78
Dispilidemia	0.9	0.3	2.87	1.36 - 6.03	0.8	0.5	1.58	0.51 - 4.94
Previous Cesarean Section	26.1	20.1	1.36	1.16 - 1.59	27.6	17.1	1.76	1.41 - 2.19
Previous PTB	6.4	1.6	3.10	2.32 - 4.14	8.0	1.2	5.33	3.68 - 7.72
IPI < 18 months	22.6	22.9	0.99	0.81 - 1.21	16.5	18.9	0.96	0.70 - 1.31
IPI > 59 months	18.7	14.8	1.25	1.01 - 1.54	18.6	13.1	1.50	1.11 - 2.03
< 3 prenatal care visits	14.7	4.9	2.87	2.35 - 3.50	10.3	2.4	3.83	2.77 - 5.29
Housing Insecurity	2.9	1.1	2.43	1.60 - 3.68	0.8	0.2	3.71	1.19 - 11.55
Intimate Partner Violence	0.5	0.2	2.02	0.76 - 5.40	0.3	0.0	4.93	0.69 - 36.07

Relative Risk

≤ 0.7

n/s

≥ 3.0+

Color = $P < .05$

© 2024 Jelliffe-Pawlowski LL et al. *JAMA Network Open.*

eTable 4. (continued)

Page 2 of 7

Risk/Protective Factors	Asian							
	Public Insurance				Non-Public Insurance			
	PTB %	Term %	RR	95% CI	PTB %	Term %	RR	95% CI
Age < 18 years	1.0	0.5	1.84	1.49 - 2.26	0.1	0.0	2.58	1.85 - 3.60
Age > 34 years	27.4	21.8	1.33	1.27 - 1.39	41.6	34.5	1.33	1.30 - 1.36
< 12 years education	10.0	8.3	1.17	1.08 - 1.26	1.0	0.8	1.01	0.90 - 1.13
> 12 years education	53.9	56.5	0.93	0.89 - 0.98	89.2	90.1	0.83	0.80 - 0.87
Public insurance for prenatal care	94.7	96.3	0.70	0.64 - 0.77	0.4	0.5	0.96	0.81 - 1.13
WIC participation	66.6	69.5	0.89	0.85 - 0.92	9.7	9.0	1.09	1.05 - 1.13
Nulliparous	38.0	38.9	0.97	0.93 - 1.01	49.6	48.0	1.07	1.05 - 1.09
Mom born outside the US	73.9	78.8	0.78	0.74 - 0.82	73.3	77.6	0.81	0.79 - 0.83
FIPS 2	7.6	7.0	1.14	1.05 - 1.23	13.0	12.1	1.10	1.07 - 1.14
FIPS 3	24.7	21.7	1.18	1.12 - 1.24	8.8	6.9	1.29	1.24 - 1.34
FIPS 4	3.6	3.9	0.97	0.87 - 1.08	1.2	1.1	1.16	1.05 - 1.28
FIPS 5-6	0.7	0.8	0.92	0.72 - 1.19	0.2	0.2	1.34	1.05 - 1.71
Pre-pregnancy BMI < 18.5 kg/m2	8.6	8.8	1.09	1.01 - 1.17	6.2	7.8	0.89	0.85 - 0.93
Pre-pregnancy BMI ≥ 30 kg/m2	13.4	9.8	1.48	1.39 - 1.58	10.9	6.4	1.81	1.75 - 1.88
Preexisting Diabetes	3.4	1.1	2.71	2.43 - 3.03	3.0	1.2	2.43	2.28 - 2.58
Preexisting Hypertension	7.0	1.4	4.06	3.74 - 4.39	7.9	1.7	4.05	3.89 - 4.21
Smoking	3.0	1.3	2.06	1.83 - 2.33	0.7	0.4	1.65	1.44 - 1.88
Drug/Alcohol Use	2.4	0.6	3.23	2.83 - 3.69	0.4	0.1	2.81	2.38 - 3.31
Mental Health Condition	5.4	2.3	2.17	1.99 - 2.38	5.2	3.0	1.69	1.61 - 1.77
Sickle Cell Anemia	0.0	0.0	1.53	0.22 - 10.89	0.0	0.0	1.18	0.38 - 3.65
Anemia	12.3	9.9	1.25	1.17 - 1.33	13.0	10.6	1.24	1.20 - 1.28
Gestational Diabetes	19.0	13.7	1.43	1.36 - 1.51	22.7	15.1	1.59	1.55 - 1.63
Gestational Hypertension	12.2	4.4	2.67	2.51 - 2.85	13.4	4.9	2.70	2.62 - 2.79
Infection (non-COVID-19, non-STI)	13.2	7.6	1.75	1.65 - 1.86	8.9	5.0	1.79	1.72 - 1.86
Sexually Transmitted Infection	0.5	0.5	1.08	0.81 - 1.43	0.6	0.4	1.28	1.11 - 1.48
COVID-19 (2020+ only)	2.6	1.5	1.69	1.03 - 2.77	1.4	0.7	1.88	1.36 - 2.59
Asthma	3.5	2.1	1.66	1.49 - 1.85	4.9	3.0	1.58	1.51 - 1.67
Sleep Disorder	0.2	0.1	1.75	1.09 - 2.82	0.5	0.2	2.68	2.31 - 3.11
Autoimmune Disorder	0.6	0.1	3.48	2.66 - 4.56	0.8	0.3	2.48	2.20 - 2.81
Malignancy	0.2	0.0	4.84	3.16 - 7.43	0.2	0.1	3.03	2.34 - 3.92
Dispilidemia	0.4	0.2	2.13	1.54 - 2.95	1.7	0.8	2.10	1.94 - 2.28
Previous Cesarean Section	18.6	15.6	1.22	1.15 - 1.28	17.1	15.3	1.13	1.10 - 1.16
Previous PTB	3.2	0.6	4.02	3.67 - 4.52	3.3	0.7	4.41	4.15 - 4.69
IPI < 18 months	16.6	16.9	1.12	1.05 - 1.20	10.7	11.9	1.05	1.01 - 1.10
IPI > 59 months	17.7	14.2	1.39	1.31 - 1.49	12.8	10.2	1.44	1.38 - 1.49
< 3 prenatal care visits	3.9	0.8	3.97	3.57 - 4.41	0.9	0.3	2.88	2.57 - 3.24
Housing Insecurity	0.2	0.1	2.50	1.51 - 4.15	0.0	0.0	3.49	1.57 - 7.76
Intimate Partner Violence	0.0	0.0	1.18	0.38 - 3.67	0.0	0.0	2.02	1.05 - 3.88

© 2024 Jelliffe-Pawlowski LL et al. *JAMA Network Open.*

eTable 4. (continued)

	Black							
	Public Insurance				Non-Public Insurance			
**Risk/Protective Factors**	**PTB %**	**Term %**	**RR**	**95% CI**	**PTB %**	**Term %**	**RR**	**95% CI**
Age < 18 years	2.5	2.6	1.00	0.89 - 1.12	1.1	1.0	1.15	0.94 - 1.41
Age > 34 years	16.5	11.4	1.47	1.40 - 1.54	28.4	23.2	1.28	1.22 - 1.34
< 12 years education	20.0	17.7	1.13	1.08 - 1.19	6.0	4.5	1.28	1.17 - 1.42
> 12 years education	38.0	40.2	0.96	0.92 - 1.00	67.7	69.9	0.96	0.92 - 1.01
Public insurance for prenatal care	93.0	97.0	0.47	0.44 - 0.50	2.0	2.1	0.96	0.83 - 1.12
WIC participation	74.7	80.3	0.75	0.72 - 0.78	40.9	42.1	0.96	0.92 - 1.00
Nulliparous	29.8	34.6	0.82	0.79 - 0.85	43.0	41.9	1.04	1.00 - 1.09
Mom born outside the US	5.9	10.1	0.59	0.54 - 0.63	11.3	15.0	0.74	0.69 - 0.79
FIPS 2	15.9	14.9	1.09	1.04 - 1.14	16.6	15.6	1.07	1.01 - 1.14
FIPS 3	18.4	17.3	1.09	1.04 - 1.14	10.9	10.9	1.01	0.94 - 1.08
FIPS 4	1.5	1.6	0.94	0.81 - 1.09	1.6	1.6	1.02	0.86 - 1.21
FIPS 5-6	0.3	0.3	1.00	0.71 - 1.40	0.2	0.2	1.05	0.65 - 1.68
Pre-pregnancy BMI < 18.5 kg/m2	5.1	4.1	1.27	1.17 - 1.38	2.6	2.9	1.15	1.01 - 1.30
Pre-pregnancy BMI ≥ 30 kg/m2	31.4	30.8	1.05	1.01 - 1.10	34.9	31.7	1.15	1.09 - 1.21
Preexisting Diabetes	4.2	1.3	2.69	2.47 - 2.94	4.2	1.3	2.80	2.52 - 3.11
Preexisting Hypertension	13.3	4.3	2.79	2.65 - 2.94	14.3	4.8	2.78	2.62 - 2.95
Smoking	15.6	9.7	1.61	1.54 - 1.69	5.5	3.3	1.62	1.48 - 1.78
Drug/Alcohol Use	14.9	7.5	1.94	1.85 - 2.04	6.3	3.1	1.90	1.74 - 2.07
Mental Health Condition	22.6	12.9	1.81	1.74 - 1.89	16.4	10.5	1.58	1.50 - 1.68
Sickle Cell Anemia	0.9	0.5	1.70	1.41 - 2.06	0.8	0.4	1.75	1.38 - 2.22
Anemia	24.3	19.9	1.26	1.21 - 1.31	29.4	25.3	1.21	1.15 - 1.26
Gestational Diabetes	9.7	6.8	1.40	1.32 - 1.49	11.7	7.8	1.49	1.40 - 1.60
Gestational Hypertension	17.0	8.7	1.93	1.84 - 2.03	19.9	9.9	2.05	1.94 - 2.16
Infection (non-COVID-19, non-STI)	26.2	19.2	1.43	1.37 - 1.49	19.4	12.2	1.63	1.55 - 1.72
Sexually Transmitted Infection	2.9	2.1	1.36	1.23 - 1.51	2.7	2.4	1.12	0.99 - 1.28
COVID-19 (2020+ only)	2.4	2.2	1.06	0.69 - 1.64	1.8	1.8	1.04	0.60 - 1.80
Asthma	16.8	13.3	1.27	1.21 - 1.33	14.8	11.5	1.29	1.22 - 1.37
Sleep Disorder	0.9	0.4	2.26	1.88 - 2.70	1.3	0.6	2.08	1.74 - 2.50
Autoimmune Disorder	1.1	0.4	2.58	2.18 - 3.05	1.2	0.5	2.03	1.67 - 2.48
Malignancy	0.2	0.1	3.13	2.13 - 4.60	0.2	0.1	2.95	1.88 - 4.63
Dispilidemia	0.9	0.3	2.26	2.12 - 3.09	1.9	1.0	1.84	1.57 - 2.14
Previous Cesarean Section	25.3	20.5	1.28	1.23 - 1.33	21.2	17.8	1.22	1.16 - 1.28
Previous PTB	6.3	1.6	3.07	2.85 - 3.30	5.4	1.2	3.68	3.34 - 4.04
IPI < 18 months	21.0	18.3	1.21	1.15 - 1.28	12.3	13.7	1.07	0.99 - 1.16
IPI > 59 months	17.9	15.7	1.20	1.14 - 1.27	18.1	15.8	1.33	1.24 - 1.43
< 3 prenatal care visits	8.4	2.8	2.69	2.52 - 2.86	3.8	1.0	3.27	2.93 - 3.65
Housing Insecurity	1.7	0.8	1.87	1.64 - 2.14	0.7	0.2	2.53	1.95 - 3.29
Intimate Partner Violence	0.3	0.2	1.37	1.00 - 1.89	0.2	0.1	2.25	0.76 - 2.08

© 2024 Jelliffe-Pawlowski LL et al. *JAMA Network Open.*

eTable 4. (continued)

	Hawaiian/Pacific Islander							
	Public Insurance				Non-Public Insurance			
**Risk/Protective Factors**	**PTB %**	**Term %**	**RR**	**95% CI**	**PTB %**	**Term %**	**RR**	**95% CI**
Age < 18 years	1.1	1.1	1.14	0.57 - 2.28	0.6	0.5	1.34	0.50 - 3.59
Age > 34 years	18.3	12.6	1.50	1.24 - 1.82	34.0	22.2	1.71	1.46 - 2.00
< 12 years education	13.6	11.5	1.23	0.98 - 1.54	4.3	3.5	1.13	0.77 - 1.67
> 12 years education	35.2	34.6	1.07	0.91 - 1.26	58.6	62.2	0.90	0.76 - 1.06
Public insurance for prenatal care	93.2	96.5	0.54	0.40 - 0.73	1.8	1.2	1.43	0.81 - 2.53
WIC participation	62.5	69.2	0.76	0.65 - 0.89	25.6	28.3	0.88	0.74 - 1.05
Nulliparous	32.2	28.4	1.18	1.01 - 1.39	40.8	35.0	1.25	1.08 - 1.46
Mom born outside the US	45.2	42.8	1.09	0.94 - 1.27	45.2	40.8	1.18	1.02 - 1.37
FIPS 2	17.6	19.3	0.89	0.73 - 1.09	18.0	19.2	0.94	0.77 - 1.15
FIPS 3	15.2	14.7	1.00	0.81 - 1.23	14.0	14.0	0.99	0.80 - 1.24
FIPS 4	1.7	2.2	0.77	0.43 - 1.36	2.2	2.0	1.09	0.65 - 1.83
FIPS 5-6	0.7	1.6	0.47	0.19 - 1.13	0.6	0.9	0.71	0.26 - 1.89
Pre-pregnancy BMI < 18.5 kg/m2	1.4	1.3	0.99	0.52 - 1.88	1.9	1.6	1.34	0.76 - 2.36
Pre-pregnancy BMI ≥ 30 kg/m2	46.2	50.3	0.84	0.69 - 1.02	45.1	44.9	1.11	0.91 - 1.34
Preexisting Diabetes	8.2	1.9	3.55	2.71 - 4.64	7.8	2.4	2.87	2.17 - 3.80
Preexisting Hypertension	12.9	2.8	3.94	3.16 - 3.91	15.2	3.9	3.56	2.88 - 4.39
Smoking	11.4	8.3	1.38	1.09 - 1.74	4.9	3.9	1.23	0.87 - 1.75
Drug/Alcohol Use	6.4	2.9	2.06	1.52 - 2.78	2.5	1.3	1.87	1.16 - 3.03
Mental Health Condition	13.4	6.9	1.92	1.54 - 2.38	11.4	6.8	1.66	1.31 - 2.10
Sickle Cell Anemia	0.0	0.0	n/c	n/c	0.0	0.0	n/c	n/c
Anemia	17.1	13.9	1.25	1.02 - 1.52	19.9	16.9	1.20	1.00 - 1.45
Gestational Diabetes	18.5	13.4	1.42	1.17 - 1.71	21.7	15.0	1.51	1.25 - 1.81
Gestational Hypertension	13.5	8.7	1.56	1.26 - 1.94	18.2	8.9	2.08	1.71 - 2.53
Infection (non-COVID-19, non-STI)	22.4	15.5	1.51	1.26 - 1.80	15.7	10.3	1.55	1.26 - 1.90
Sexually Transmitted Infection	1.7	1.1	1.52	0.86 - 2.69	1.5	0.8	1.84	0.99 - 3.44
COVID-19 (2020+ only)	2.2	2.3	0.97	0.13 - 7.07	0.0	3.1	n/c	n/c
Asthma	7.4	5.7	1.29	0.97 - 1.71	8.6	6.9	1.24	0.95 - 1.63
Sleep Disorder	0.3	0.3	1.03	0.26 - 4.13	1.2	0.5	2.26	1.13 - 4.53
Autoimmune Disorder	0.9	0.3	2.65	1.18 - 5.91	1.5	0.3	3.49	1.87 - 6.52
Malignancy	0.1	0.1	1.69	0.24 - 12.04	0.2	0.0	4.03	0.57 - 28.67
Dispilidemia	1.0	0.3	2.61	1.24 - 5.49	1.3	1.2	1.12	0.58 - 2.17
Previous Cesarean Section	23.4	19.1	1.27	1.07 - 1.51	20.8	18.3	1.16	0.96 - 1.39
Previous PTB	4.7	1.1	3.61	2.53 - 51.4	4.4	1.3	3.37	2.32 - 4.88
IPI < 18 months	29.7	28.7	1.38	1.12 - 1.72	14.3	20.9	0.87	0.67 - 1.13
IPI > 59 months	12.8	11.0	1.54	1.18 - 2.00	18.5	13.2	1.68	1.32 - 2.13
< 3 prenatal care visits	10.8	3.8	2.68	2.11 - 3.40	4.1	1.5	2.54	1.74 - 3.70
Housing Insecurity	1.1	0.3	2.81	1.40 - 5.64	0.3	0.1	2.69	0.67 - 10.78
Intimate Partner Violence	0.1	0.1	1.98	0.28 - 14.05	0.2	0.1	2.01	0.28 - 14.33

© 2024 Jelliffe-Pawlowski LL et al. *JAMA Network Open.*

eTable 4. (continued)

	Hispanic							
	Public Insurance				Non-Public Insurance			
**Risk/Protective Factors**	**PTB %**	**Term %**	**RR**	**95% CI**	**PTB %**	**Term %**	**RR**	**95% CI**
Age < 18 years	3.0	3.0	1.08	1.04 - 1.12	1.5	1.2	1.37	1.28 - 1.47
Age > 34 years	19.8	13.8	1.50	1.47 - 1.52	25.7	20.2	1.34	1.31 - 1.36
< 12 years education	38.7	37.5	1.06	1.04 - 1.07	11.1	8.9	1.20	1.17 - 1.24
> 12 years education	23.5	24.2	1.00	0.98 - 1.02	58.7	61.7	0.94	0.92 - 0.96
Public insurance for prenatal care	95.3	97.5	0.55	0.53 - 0.56	2.0	2.0	0.99	0.94 - 1.06
WIC participation	82.6	86.6	0.75	0.74 - 0.77	38.6	38.5	1.00	0.99 - 1.02
Nulliparous	28.7	30.6	0.92	0.91 - 0.93	39.5	39.3	1.01	0.99 - 1.02
Mom born outside the US	10.3	9.4	1.06	1.03 - 1.08	7.2	6.5	1.09	1.06 - 1.13
FIPS 2	10.2	10.5	0.96	0.94 - 0.98	12.9	12.7	1.01	0.98 - 1.03
FIPS 3	25.5	25.7	0.98	0.97 - 0.99	18.3	18.3	0.99	0.97 - 1.01
FIPS 4	4.9	5.2	0.93	0.90 - 0.96	4.2	4.4	0.96	0.92 - 1.00
FIPS 5-6	1.0	1.1	0.92	0.7 - 0.98	0.6	0.7	0.85	0.76 - 0.94
Pre-pregnancy BMI < 18.5 kg/m2	2.7	2.3	1.25	1.20 - 1.30	2.1	1.9	1.29	1.21 - 1.37
Pre-pregnancy BMI ≥ 30 kg/m2	33.1	30.0	1.19	1.17 - 1.21	34.1	28.9	1.34	1.31 - 1.36
Preexisting Diabetes	4.4	1.3	2.99	2.90 - 3.08	4.2	1.4	2.78	2.67 - 2.90
Preexisting Hypertension	6.4	1.5	3.63	3.54 - 3.73	7.2	2.1	3.12	3.02 - 3.22
Smoking	2.9	1.5	1.81	1.75 - 1.88	1.5	0.8	1.71	1.60 - 1.83
Drug/Alcohol Use	4.8	1.8	2.50	2.43 - 2.57	2.4	1.0	2.28	2.16 - 2.41
Mental Health Condition	8.2	4.1	1.95	1.91 - 2.00	9.9	6.6	1.48	1.44 - 1.52
Sickle Cell Anemia	0.0	0.0	2.28	1.52 - 3.4	0.0	0.0	1.58	0.79 - 3.17
Anemia	13.3	11.0	1.22	1.19 - 1.24	16.0	14.1	1.15	1.12 - 1.17
Gestational Diabetes	14.4	9.6	1.52	1.49 - 1.54	14.7	10.1	1.48	1.44 - 1.51
Gestational Hypertension	15.5	5.9	2.62	2.57 - 2.66	16.8	6.8	2.50	2.45 - 2.56
Infection (non-COVID-19, non-STI)	15.7	10.5	1.52	1.49 - 1.55	13.0	8.2	1.61	1.57 - 1.65
Sexually Transmitted Infection	1.1	0.7	1.49	1.41 - 1.59	1.0	0.9	1.11	1.02 - 1.21
COVID-19 (2020+ only)	5.3	3.8	1.38	1.25 - 1.53	3.7	2.7	1.33	1.15 - 1.55
Asthma	5.1	3.7	1.37	1.33 - 1.40	6.8	5.7	1.19	1.15 - 1.23
Sleep Disorder	0.3	0.1	2.34	2.09 - 2.62	0.6	0.3	1.86	1.67 - 2.08
Autoimmune Disorder	0.6	0.2	2.73	2.51 - 2.96	1.0	0.4	2.21	2.03 - 2.41
Malignancy	0.2	0.0	3.66	3.16 - 4.23	0.2	0.1	2.73	2.25 - 3.31
Dispilidemia	0.6	0.2	2.54	2.34 - 2.76	1.9	1.0	1.83	1.72 - 1.94
Previous Cesarean Section	25.3	18.9	1.41	1.39 - 1.43	20.6	16.4	1.29	1.26 - 1.32
Previous PTB	4.1	0.8	4.04	3.91 - 4.17	4.2	0.9	4.00	3.84 - 4.18
IPI < 18 months	16.9	16.0	1.19	1.16 - 1.21	13.1	13.8	1.08	1.05 - 1.12
IPI > 59 months	23.0	19.7	1.30	1.27 - 1.32	19.4	16.4	1.33	1.30 - 1.36
< 3 prenatal care visits	4.6	1.2	3.27	3.18 - 3.37	3.0	0.8	3.25	3.10 - 3.42
Housing Insecurity	0.4	0.1	3.08	2.80 - 3.38	0.2	0.0	3.60	2.93 - 4.42
Intimate Partner Violence	0.1	0.0	1.49	1.17 - 1.91	0.1	0.0	2.02	1.47 - 2.77

© 2024 Jelliffe-Pawlowski LL et al. *JAMA Network Open.*

eTable 4. (continued)

	White							
	Public Insurance				Non-Public Insurance			
**Risk/Protective Factors**	**PTB %**	**Term %**	**RR**	**95% CI**	**PTB %**	**Term %**	**RR**	**95% CI**
Age < 18 years	1.4	1.3	1.15	1.02 - 1.30	0.3	0.2	1.68	1.42 - 2.00
Age > 34 years	17.8	13.1	1.40	1.35 - 1.45	33.1	28.9	1.21	1.18 - 1.23
< 12 years education	15.3	12.5	1.20	1.15 - 1.25	1.7	1.1	1.37	1.28 - 1.48
> 12 years education	44.6	48.3	0.92	0.90 - 0.95	82.8	85.5	0.85	0.83 - 0.87
Public insurance for prenatal care	91.1	94.8	0.49	0.46 - 0.51	0.7	0.6	1.14	1.02 - 1.28
WIC participation	61.1	65.2	0.85	0.82 - 0.87	9.9	8.8	1.14	1.10 - 1.16
Nulliparous	35.0	35.3	0.95	0.92 - 0.98	50.2	45.6	1.19	1.17 - 1.21
Mom born outside the US	14.0	17.8	0.76	0.73 - 0.80	12.5	13.7	0.90	0.88 - 0.93
FIPS 2	12.7	12.8	1.02	0.97 - 1.06	14.6	14.7	1.01	0.98 - 1.04
FIPS 3	27.2	26.3	1.05	1.02 - 1.09	18.5	17.8	1.05	1.02 - 1.07
FIPS 4	10.2	10.0	1.04	0.99 - 1.09	4.0	4.1	1.00	0.95 - 1.04
FIPS 5-6	81.9	8.5	0.99	0.94 - 1.04	2.7	2.7	1.00	0.95 - 1.06
Pre-pregnancy BMI < 18.5 kg/m2	6.4	4.7	1.42	1.33 - 1.50	4.0	3.4	1.30	1.24 - 1.36
Pre-pregnancy BMI ≥ 30 kg/m2	25.0	24.4	1.08	1.04 - 1.12	20.1	15.7	1.39	1.36 - 1.43
Preexisting Diabetes	3.3	0.9	3.17	2.93 - 3.43	2.6	0.7	3.14	2.97 - 3.33
Preexisting Hypertension	7.2	2.2	2.96	2.81 - 3.13	6.5	2.0	3.00	2.89 - 3.11
Smoking	23.5	14.8	1.69	1.64 - 1.75	3.6	1.9	1.82	1.74 - 1.91
Drug/Alcohol Use	17.8	7.1	2.55	2.46 - 2.65	2.6	1.0	2.38	2.25 - 2.52
Mental Health Condition	26.5	14.7	1.96	1.89 - 2.02	14.2	9.2	1.58	1.54 - 1.62
Sickle Cell Anemia	0.0	0.0	2.89	0.93 - 8.97	0.0	0.0	1.46	0.47 - 4.53
Anemia	13.7	10.2	1.36	1.31 - 1.42	12.2	9.5	1.31	1.27 - 1.35
Gestational Diabetes	10.8	7.7	1.41	1.35 - 1.47	10.8	7.0	1.57	1.52 - 1.61
Gestational Hypertension	14.1	6.5	2.18	2.09 - 2.27	16.5	7.0	2.44	2.38 - 2.50
Infection (non-COVID-19, non-STI)	19.8	13.1	1.58	1.52 - 1.63	10.6	6.2	1.73	1.68 - 1.78
Sexually Transmitted Infection	1.9	1.1	1.57	1.42 - 1.74	1.1	1.1	1.02	0.93 - 1.11
COVID-19 (2020+ only)	2.2	1.7	1.30	0.92 - 1.82	1.5	0.9	1.62	1.24 - 2.11
Asthma	11.5	8.1	1.42	1.36 - 1.48	8.0	6.0	1.33	1.29 - 1.38
Sleep Disorder	0.8	0.3	2.24	1.91 - 2.63	0.9	0.3	2.45	2.22 - 2.70
Autoimmune Disorder	1.0	0.5	2.05	1.78 - 2.37	1.3	0.8	1.66	1.53 - 1.79
Malignancy	0.2	0.1	2.73	2.01 - 3.69	0.3	0.1	3.17	2.69 - 3.72
Dispilidemia	0.7	0.3	2.42	2.03 - 2.87	1.1	0.6	1.78	1.63 - 1.94
Previous Cesarean Section	22.3	17.1	1.36	1.31 - 1.31	17.9	14.2	1.30	1.27 - 1.33
Previous PTB	5.2	1.0	4.07	3.81 - 4.34	4.4	0.9	4.78	4.57 - 5.00
IPI < 18 months	18.7	17.9	1.19	1.14 - 1.24	13.6	16.4	0.99	0.96 - 1.03
IPI > 59 months	16.2	13.5	1.36	1.30 - 1.42	8.3	6.6	1.47	1.42 - 1.53
< 3 prenatal care visits	9.5	2.2	3.89	3.71 - 4.08	1.5	0.3	4.52	4.20 - 4.87
Housing Insecurity	2.0	0.6	3.00	2.71 - 3.33	0.2	0.0	4.18	3.35 - 5.22
Intimate Partner Violence	0.2	0.1	2.02	1.46 - 2.80	0.0	0.0	1.27	0.66 - 2.43

© 2024 Jelliffe-Pawlowski LL et al. *JAMA Network Open.*

eTable 4. (continued)

Page 7 of 7

	Other							
	Public Insurance				Non-Public Insurance			
**Risk/Protective Factors**	**PTB %**	**Term %**	**RR**	**95% CI**	**PTB %**	**Term %**	**RR**	**95% CI**
Age < 18 years	2.1	2.3	0.99	0.84 - 1.18	0.8	0.5	1.66	1.32 - 2.07
Age > 34 years	18.7	12.9	1.49	1.39 - 1.58	35.7	30.3	1.26	1.21 - 1.31
< 12 years education	9.4	8.8	1.09	0.99 - 1.20	1.5	1.1	1.24	1.04 - 1.47
> 12 years education	24.6	27.2	0.94	0.88 - 1.01	40.2	44.1	0.86	0.80 - 0.93
Public insurance for prenatal care	90.9	96.5	0.42	0.38 - 0.46	0.9	0.8	1.08	0.87 - 1.33
WIC participation	61.6	69.3	0.73	0.70 - 0.77	13.9	13.3	1.06	1.00 - 1.12
Nulliparous	32.5	36.4	0.86	0.81 - 0.90	48.7	47.4	1.05	1.01 - 1.10
Mom born outside the US	14.6	15.9	0.92	0.86 - 0.98	21.7	21.3	1.03	0.98 - 1.08
FIPS 2	14.6	14.9	1.00	0.93 - 1.07	14.5	14.2	1.03	0.98 - 1.09
FIPS 3	20.5	19.4	1.06	1.00 - 1.13	14.3	14.9	0.97	0.92 - 1.03
FIPS 4	5.3	5.0	1.06	0.95 - 1.19	2.4	2.1	1.16	1.02 - 1.31
FIPS 5-6	3.1	3.9	0.82	0.71 - 0.94	1.3	1.2	1.10	0.93 - 1.31
Pre-pregnancy BMI < 18.5 kg/m2	4.9	3.8	1.35	1.20 - 1.52	3.6	3.5	1.17	1.05 - 1.30
Pre-pregnancy BMI ≥ 30 kg/m2	28.3	27.4	1.11	1.04 - 1.18	23.8	18.4	1.47	1.40 - 1.55
Preexisting Diabetes	4.3	1.2	2.96	2.63 - 3.34	3.6	1.1	2.88	2.59 - 3.19
Preexisting Hypertension	9.0	2.5	3.10	2.85 - 3.38	9.0	2.5	3.22	3.01 - 3.45
Smoking	15.9	10.0	1.61	1.50 - 1.72	3.6	1.9	1.80	1.62 - 2.00
Drug/Alcohol Use	16.7	7.0	2.37	2.22 - 2.53	3.9	1.6	2.28	2.06 - 2.52
Mental Health Condition	25.3	14.5	1.85	1.74 - 1.96	16.4	10.5	1.60	1.51 - 1.68
Sickle Cell Anemia	0.2	0.1	1.60	0.86 - 2.98	0.1	0.0	2.86	1.43 - 5.72
Anemia	18.7	14.1	1.36	1.27 - 1.45	16.2	13.4	1.23	1.17 - 1.30
Gestational Diabetes	11.7	9.1	1.29	1.19 - 1.39	14.4	9.6	1.53	1.45 - 1.62
Gestational Hypertension	15.7	7.9	1.99	1.86 - 2.12	17.1	8.1	2.15	2.04 - 2.26
Infection (non-COVID-19, non-STI)	20.0	13.9	1.49	1.40 - 1.58	11.8	7.0	1.70	1.60 - 1.80
Sexually Transmitted Infection	2.4	1.8	1.36	1.16 - 1.59	1.9	1.6	1.17	1.02 - 1.36
COVID-19 (2020+ only)	4.1	2.8	1.42	0.98 - 2.08	2.5	1.5	1.67	1.14 - 2.45
Asthma	12.5	9.9	1.26	1.17 - 1.36	10.1	8.0	1.26	1.18 - 1.34
Sleep Disorder	1.2	0.4	2.78	2.22 - 3.49	1.3	0.4	2.54	2.13 - 3.03
Autoimmune Disorder	0.9	0.4	1.97	1.51 - 2.56	1.3	0.6	1.99	1.68 - 2.37
Malignancy	0.2	0.1	2.73	1.55 - 4.82	0.3	0.1	2.80	1.93 - 4.05
Dispilidemia	0.9	0.3	2.70	2.09 - 3.49	1.6	0.8	1.79	1.53 - 2.10
Previous Cesarean Section	23.0	17.0	1.40	1.33 - 1.49	18.0	14.1	1.31	1.24 - 1.38
Previous PTB	5.0	1.2	3.35	2.99 - 3.75	3.9	0.9	3.79	3.42 - 4.20
IPI < 18 months	18.8	17.3	1.24	1.15 - 1.34	12.7	14.0	1.07	1.00 - 1.15
IPI > 59 months	18.7	15.7	1.36	1.26 - 1.47	12.9	9.8	1.53	1.42 - 1.64
< 3 prenatal care visits	10.3	2.9	3.19	2.95 - 3.46	2.7	0.5	4.36	3.86 - 4.91
Housing Insecurity	2.3	0.7	2.68	2.28 - 3.16	0.4	0.1	4.01	2.93 - 5.49
Intimate Partner Violence	0.2	0.2	0.95	0.51 - 1.76	0.1	0.1	2.04	1.13 - 3.79

Abbreviations: BMI, body mass index; CI, confidence interval; FIPS, Federal Information Processing Standard county code where 1 = most urban to 6 = most rural;[3] IPI, interpregnancy interval; n/c, not calculated/ displayed when n < 11 per state requirements (to protect individual-level privacy); n/s, non-significant; PTB, preterm birth (gestational weeks < 37 completed weeks); RR, relative risk; STI, sexually transmitted infection; WIC, Nutritional program for Women, Infants, and Children[4]

[a]All comparisons yes versus no except for: Age where referent = 18-34 years, education = 12 years, FIPS (most urban) = 1, BMI = 18.5-29.9, IPI = 18-59 months

[b]See more complete coding for risk and protective factors in eTable 1

© 2024 Jelliffe-Pawlowski LL et al. *JAMA Network Open.*

eTable 5. Pattern Over Time of Risk and Protective Factors for Preterm Birth (PTB) (by Percent) With Test for Trend: California Singleton Births by Insurance and Racial and Ethnic Groups From 2011 to 2022 (Presented for Factors Where Significant in eTable 4)

	Public Insurance											
	2011	2012	2013	2014	2015	2016	2017	2018	2019	2020	2021	Trend
**American Indian/ Alaska Native**												*P* =
**Protective Factors** (RR < 1.00, *P* < .05)												
Public insurance for prenatal care	97.3	97.0	96.1	94.1	94.4	93.6	94.1	94.8	93.3	93.8	91.9	< 0.001
WIC participation	81.1	81.1	79.1	77.3	74.0	69.3	69.0	65.3	62.0	58.0	53.0	< 0.001
Nulliparous	31.4	29.9	32.7	27.9	36.6	25.1	23.9	24.2	25.8	25.7	24.7	< 0.001
**Risk Factors** (RR > 1.00, P < .05)												
Age > 34 years	8.2	10.3	8.8	9.8	10.6	12.9	13.2	15.4	14.3	16.3	17.3	< 0.001
< 12 years education	28.4	28.0	22.9	25.7	22.1	23.2	19.2	19.3	19.4	19.2	17.1	< 0.001
Preexisting Diabetes	1.2	2.0	1.9	1.4	1.2	3.0	1.5	3.8	2.9	3.0	4.3	< 0.001
Preexisting Hypertension	2.8	3.5	3.8	3.6	4.2	5.1	4.3	3.8	2.9	3.7	6.0	0.047
Smoking	17.6	19.5	18.4	21.3	17.4	17.9	17.8	16.9	17.3	13.7	12.7	< 0.001
Drug/Alcohol Use	10.0	10.1	11.8	12.5	12.2	15.9	16.3	13.8	11.7	11.7	13.8	0.005
Mental Health Condition	12.2	14.3	15.7	16.1	16.9	25.2	26.7	24.8	25.2	21.7	25.9	< 0.001
Anemia	9.3	14.0	14.4	17.9	13.2	12.5	15.2	11.3	9.5	16.5	17.3	0.094
Gestational Diabetes	8.9	10.8	11.2	11.4	11.4	10.6	13.2	6.5	6.0	9.3	9.7	0.037
Gestational Hypertension	6.1	8.8	9.1	8.8	7.8	8.8	10.9	8.4	7.5	10.2	10.4	0.025
Infection (non-STI, non-COVID-19)	12.1	15.3	15.8	17.6	21.6	20.8	18.5	15.1	13.9	13.5	17.3	0.387
Sexually Transmitted Infection	0.1	0.7	0.5	0.6	0.9	4.3	4.0	2.8	3.4	3.0	6.2	< 0.001
Asthma	12.1	11.2	10.5	10.6	10.1	13.3	11.8	11.0	11.0	13.0	11.4	0.571
Sleep Disorder	0.0	0.4	0.0	0.1	1.0	1.0	0.4	0.4	0.3	0.7	0.7	0.022
Dispilidemia	0.0	0.0	0.3	0.2	0.6	0.7	0.3	0.4	0.2	0.3	0.5	0.069
Previous Cesarean Section	19.4	20.1	19.4	21.9	21.2	19.4	23.6	21.5	19.1	22.5	20.4	0.288
Previous PTB	1.5	2.2	1.8	1.7	1.6	2.1	2.7	1.5	2.0	3.7	2.6	0.038
IPI > 59 months	9.9	12.3	11.7	16.5	14.4	16.6	18.8	18.9	18.5	18.0	15.2	< 0.001
< 3 of prenatal care visits	3.4	3.2	5.3	5.4	6.1	6.8	6.1	7.0	6.4	8.2	8.6	< 0.001
Housing insecurity	0.7	0.3	0.6	0.9	1.2	1.7	1.8	1.6	1.2	1.3	3.3	< 0.001
	**Non-Public Insurance**											
**Protective Factors** (RR < 1.00, *P* < .05)												
FIPS 5-6	12.4	12.8	15.5	16.2	16.9	21.3	17.4	23.6	22.5	26.8	25.4	< 0.001
**Risk Factors** (RR > 1.00, P < .05)												
Age > 34 years	18.4	18.0	19.7	16.2	17.1	22.3	23.9	22.2	21.3	22.3	22.6	< 0.001
< 12 years education	13.1	10.8	9.7	8.5	7.6	5.4	7.9	5.2	7.7	7.1	4.0	< 0.001
Preexisting Diabetes	1.1	1.5	1.1	1.4	0.4	2.2	2.0	2.3	2.8	3.5	3.8	< 0.001
Preexisting Hypertension	2.3	3.8	2.4	3.4	3.7	5.4	5.1	3.5	2.2	1.7	3.1	0.913
Smoking	4.1	6.2	7.7	8.8	5.8	7.2	6.9	7.5	5.3	6.7	6.1	0.664
Drug/Alcohol Use	3.4	2.4	6.1	6.1	6.9	5.4	5.9	6.8	5.3	7.1	6.6	0.001
Mental Health Condition	5.7	5.5	10.0	9.5	12.0	12.1	14.6	14.5	16.6	20.1	20.7	< 0.001
Sickle Cell Anemia	0.0	0.0	0.2	0.0	0.0	0.2	0.0	0.0	0.0	0.0	0.0	0.603
Anemia	11.4	13.3	12.9	18.5	16.0	11.7	11.4	10.8	9.1	13.6	15.5	0.474

Factor Decreasing	(green)
Factor Increasing	(orange)

Color = *P* < .05

© 2024 Jelliffe-Pawlowski LL et al. *JAMA Network Open.*

eTable 5. (continued)

	Non-Public Insurance (Continued)											
	**2011**	**2012**	**2013**	**2014**	**2015**	**2016**	**2017**	**2018**	**2019**	**2020**	**2021**	**Trend**
**American Indian/ Alaska Native** (Continued)												*P* =
**Risk Factors** (RR > 1.00, P < .05) (Continued)												
Gestational Diabetes	11.9	12.3	10.3	12.6	10.0	12.2	15.0	9.1	8.7	11.9	12.0	0.570
Gestational Hypertension	7.0	9.2	8.5	8.7	8.6	8.7	11.2	7.0	8.5	13.0	11.8	0.006
Infection (non-STI, non-COVID-19)	10.0	12.5	8.1	10.0	10.4	13.0	10.9	7.7	8.5	7.3	9.2	0.062
Asthma	7.5	6.7	6.1	7.7	8.1	7.6	10.1	9.3	7.3	11.0	7.5	0.028
Malignancy	0.2	0.0	0.0	0.0	0.2	0.2	0.2	0.0	0.2	0.0	0.0	0.973
Previous Cesarean Section	17.6	16.9	17.9	18.2	16.3	20.6	18.0	15.7	18.6	18.4	17.9	0.712
Previous PTB	1.1	1.4	2.4	0.9	1.6	1.7	1.8	1.7	1.8	2.4	2.1	0.144
IPI > 59 months	13.5	10.9	13.9	10.2	14.4	14.8	13.4	13.3	16.6	14.9	12.9	0.082
< 3 of prenatal care visits	2.8	2.1	4.4	3.6	3.0	2.2	2.4	2.7	3.2	2.6	3.1	0.744
Housing insecurity	0.2	0.0	0.2	0.2	0.0	0.0	0.0	0.4	0.0	1.1	0.5	0.009
	**Public Insurance**											
**Asian**												
**Protective Factors** (RR < 1.00, *P* < .05)												
> 12 years education	53.9	52.8	54.4	56.0	56.0	56.7	58.9	58.5	59.0	58.2	58.4	< 0.001
Public insurance for prenatal care	96.1	96.4	96.5	96.1	96.0	96.2	96.8	97.0	95.3	95.2	95.8	0.004
WIC participationb	78.1	78.3	76.4	73.4	71.9	69.4	65.4	64.0	57.7	56.4	56.1	< 0.001
Mom born outside the US	78.7	79.5	79.5	78.8	78.4	79.7	80.1	78.8	77.9	74.9	72.6	< 0.001
**Risk Factors** (RR > 1.00, P < .05)												
Age < 18 years	1.2	0.9	0.8	0.6	0.5	0.4	0.4	0.3	0.3	0.3	0.2	< 0.001
Age > 34 years	19.3	19.9	20.5	20.7	20.8	21.6	23.5	24.4	24.6	26.6	27.3	< 0.001
< 12 years education	11.5	10.5	9.9	8.4	7.5	7.6	6.5	7.4	8.0	7.2	6.0	< 0.001
FIPS 2	6.6	6.4	7.2	7.1	6.9	6.9	7.2	7.3	7.5	6.9	7.3	0.0022
FIPS 3	20.9	20.1	20.7	20.9	21.5	21.9	21.5	22.8	22.9	25.1	25.9	< 0.001
Pre-pregnancy BMI < 18.5 kg/m^2	9.7	9.7	9.4	9.4	9.1	9.8	9.1	8.0	7.6	6.5	5.9	< 0.001
Pre-pregnancy BMI ≥30 kg/m^2	7.5	8.0	8.7	9.3	9.3	9.2	10.4	11.2	12.8	14.0	15.1	< 0.001
Preexisting Diabetes	0.6	0.6	0.5	0.6	1.0	0.9	0.9	2.4	2.6	2.8	3.2	< 0.001
Preexisting Hypertension	1.4	1.5	1.5	1.7	1.8	2.0	2.1	1.8	2.0	2.5	2.6	< 0.001
Smoking	2.0	1.7	1.4	1.5	1.7	1.3	1.4	1.2	1.4	1.2	1.1	< 0.001
Drug/Alcohol Use	0.8	0.7	0.6	0.6	0.6	0.9	1.1	0.8	0.8	0.8	0.8	0.004
Mental Health Condition	1.5	1.4	1.4	1.6	1.9	2.9	3.4	3.2	3.6	4.7	4.9	< 0.001
Anemia	9.1	9.2	9.9	11.3	11.0	9.4	11.2	8.8	8.5	10.7	12.0	< 0.001
Gestational Diabetes	12.3	12.9	13.8	15.3	14.7	15.7	16.7	11.0	11.8	14.8	16.7	< 0.001
Gestational Hypertension	3.9	4.1	4.3	4.5	4.6	5.0	6.2	4.5	5.0	6.4	7.4	< 0.001
Infection (non-STI, non-COVID-19)	5.2	6.4	6.5	7.4	9.7	13.6	10.3	6.6	6.2	7.2	8.6	< 0.001
Asthma	2.0	2.0	1.9	2.1	2.3	2.0	2.3	2.1	2.1	2.8	2.8	< 0.001
Sleep Disorder	0.1	0.1	0.1	0.1	0.1	0.1	0.1	0.1	0.1	0.2	0.3	< 0.001
Autoimmune Disorder	0.1	0.2	0.2	0.2	0.2	0.2	0.2	0.1	0.1	0.1	0.2	0.583
Malignancy	0.0	0.0	0.1	0.1	0.1	0.1	0.1	0.1	0.1	0.0	0.0	0.567
Dispilidemia	0.0	0.1	0.1	0.1	0.2	0.2	0.3	0.2	0.3	0.3	0.5	< 0.001

© 2024 Jelliffe-Pawlowski LL et al. *JAMA Network Open.*

eTable 5. (continued)

	Public Insurance (Continued)											
	**2011**	**2012**	**2013**	**2014**	**2015**	**2016**	**2017**	**2018**	**2019**	**2020**	**2021**	**Trend**
**Asian** (Continued)												*P* =
**Risk Factors** (RR > 1.00, P < .05) (Continued)												
Previous Cesarean Section	13.6	14.4	14.4	15.4	15.4	16.5	17.5	16.3	17.2	17.2	17.8	< 0.001
Previous PTB	0.5	0.5	0.8	1.0	0.8	0.7	0.7	0.7	0.9	1.3	1.3	< 0.001
IPI < 18 months	16.4	16.9	16.9	17.2	16.9	17.4	16.8	16.8	16.5	16.7	16.6	0.740
IPI > 59 months	12.9	13.8	13.7	14.0	14.3	14.3	14.5	15.4	16.4	16.8	14.7	< 0.001
< 3 of prenatal care visits	1.2	1.0	1.0	1.1	0.9	1.1	0.8	1.1	1.1	1.1	1.0	0.558
Housing insecurity	0.1	0.0	0.0	0.1	0.1	0.1	0.1	0.1	0.0	0.1	0.1	0.024
	**Non-Public Insurance**											
**Protective Factors** (RR < 1.00, *P* < .05)												
> 12 years education	88.8	88.4	88.7	89.8	90.0	90.4	90.9	91.2	91.2	90.9	90.5	< 0.001
Mom born outside the US	79.9	80.2	79.7	80.6	78.6	79.0	77.9	76.1	75.6	71.2	67.0	< 0.001
Pre-pregnancy BMI < 18.5 kg/m^2	7.7	7.7	7.9	8.6	8.3	9.0	8.2	8.0	7.5	5.9	5.1	< 0.001
**Risk Factors** (RR > 1.00, P < .05)												
Age < 18 years	0.1	0.1	0.1	0.1	0.1	0.0	0.0	0.0	0.0	0.0	0.0	< 0.001
Age > 34 years	31.9	32.1	33.3	32.7	34.1	33.7	35.3	36.7	37.0	40.2	41.1	< 0.001
FIPS 2	12.0	11.2	11.4	11.8	12.0	12.7	12.9	12.5	12.3	12.9	12.7	< 0.001
FIPS 3	7.9	7.0	6.9	6.1	6.4	6.3	6.4	6.7	7.3	8.6	9.2	< 0.001
FIPS 4	1.3	1.2	1.1	1.0	1.1	1.0	0.9	1.0	1.0	1.1	1.3	< 0.001
FIPS 5-6	0.1	0.1	0.1	0.1	0.1	0.2	0.1	0.1	0.2	0.2	0.2	0.015
Pre-pregnancy BMI ≥30 kg/m^2 [b]	5.5	5.5	5.6	5.3	5.9	6.2	6.8	7.4	7.8	9.3	10.0	< 0.001
Preexisting Diabetes	0.6	0.6	0.6	0.6	0.7	1.0	1.0	2.2	2.3	2.7	2.7	< 0.001
Preexisting Hypertension	1.9	1.7	1.7	1.7	1.9	2.2	2.2	1.9	2.0	2.7	3.5	< 0.001
Smoking	0.6	0.6	0.5	0.4	0.4	0.4	0.4	0.3	0.3	0.3	0.3	< 0.001
Drug/Alcohol Use	0.1	0.1	0.1	0.1	0.1	0.2	0.2	0.2	0.2	0.2	0.2	0.004
Mental Health Condition	1.2	1.3	1.4	1.5	2.1	3.0	3.9	3.7	4.1	6.3	7.9	< 0.001
Anemia	10.1	9.8	10.4	10.8	11.5	10.1	11.0	9.7	10.2	11.6	13.6	< 0.001
Gestational Diabetes	15.2	16.1	15.9	15.9	16.7	15.7	17.1	13.1	13.5	15.2	17.1	< 0.001
Gestational Hypertension	4.2	3.9	4.2	4.2	4.8	4.9	5.9	5.6	6.2	8.1	9.6	< 0.001
Infection (non-STI, non-COVID-19)	3.4	4.0	3.8	4.0	6.6	11.1	7.3	4.2	3.5	3.9	4.2	< 0.001
Sexually Transmitted Infection	0.0	0.0	0.0	0.1	0.2	0.7	0.8	0.7	0.7	0.9	1.0	< 0.001
Asthma	2.5	2.5	2.6	2.6	3.1	3.1	3.4	3.0	3.3	4.2	4.7	< 0.001
Sleep Disorder	0.1	0.1	0.1	0.1	0.2	0.2	0.2	0.2	0.3	0.4	0.4	< 0.001
Autoimmune Disorder	0.3	0.3	0.3	0.3	0.4	0.3	0.3	0.3	0.3	0.3	0.4	0.070
Malignancy	0.0	0.1	0.1	0.1	0.1	0.1	0.1	0.1	0.0	0.1	0.1	0.877
Dispilidemia	0.5	0.6	0.7	0.8	0.9	0.8	0.9	0.9	0.8	1.1	1.4	< 0.001
Previous Cesarean Section	14.6	15.1	16.5	16.7	16.2	16.1	15.6	14.7	14.8	14.1	13.8	< 0.001
Previous PTB	0.7	0.7	0.8	0.7	0.7	0.8	0.8	0.7	0.9	1.1	1.4	< 0.001
IPI < 18 months	12.7	12.3	12.3	12.2	11.5	11.8	11.3	11.6	11.4	11.4	11.4	< 0.001
IPI > 59 months	9.5	9.7	10.9	11.2	10.6	10.1	9.8	11.2	11.0	11.1	8.5	0.319
< 3 of prenatal care visits	0.2	0.2	0.3	0.3	0.3	0.3	0.5	0.3	0.4	0.3	0.2	< 0.001
Housing insecurity	0.0	0.0	0.0	0.0	0.0	0.0	0.0	0.0	0.0	0.0	0.0	0.024

© 2024 Jelliffe-Pawlowski LL et al. *JAMA Network Open.*

eTable 5. (continued)

	**Non-Public Insurance** (Continued)											
	**2011**	**2012**	**2013**	**2014**	**2015**	**2016**	**2017**	**2018**	**2019**	**2020**	**2021**	**Trend**
**Asian** (Continued)												*P* =
**Risk Factors** (RR > 1.00, P < .05) (Continued)												
Intimate Partner Volence	0.0	0.0	0.0	0.0	0.0	0.0	0.0	0.0	0.0	0.0	0.0	< 0.001
WIC participation	11.2	11.0	10.9	10.7	10.9	8.4	8.3	7.4	6.3	6.2	6.0	< 0.001
Nulliparous	46.9	47.7	47.1	47.3	47.0	48.1	48.4	49.4	49.6	49.6	49.4	0.024
	**Public Insurance**											
**Black**												
**Protective Factors** (RR < 1.00, *P* < .05)												
Public insurance for prenatal care	97.6	97.1	97.1	96.7	96.9	96.5	95.9	95.8	96.0	95.8	95.9	< 0.001
WIC participation	86.3	86.2	85.5	84.9	82.6	79.7	76.5	75.8	73.2	67.7	64.1	< 0.001
Nulliparous	38.5	36.0	35.5	34.9	33.9	32.1	32.1	33.3	32.2	31.9	31.5	< 0.001
Mom born outside the US	7.8	8.4	8.9	9.0	10.1	10.8	11.1	10.9	10.8	10.0	10.6	< 0.001
**Risk Factors** (RR > 1.00, P < .05)												
Age > 34 years	8.9	9.6	9.9	10.4	11.4	12.8	12.7	13.8	15.4	15.2	15.5	< 0.001
< 12 years education	21.9	21.5	20.9	19.3	17.9	17.0	15.8	15.8	14.9	13.8	12.0	< 0.001
FIPS 2	13.8	14.3	15.9	15.0	14.8	15.0	15.3	16.4	16.6	14.4	14.5	0.002
FIPS 3	16.0	16.3	16.7	17.9	17.7	17.7	17.6	17.9	18.0	18.7	18.6	< 0.001
Pre-pregnancy BMI < 18.5 kg/m^2	3.9	4.3	4.4	4.4	4.4	4.3	4.5	4.4	3.8	3.7	3.5	< 0.001
Pre-pregnancy BMI ≥30 kg/m^2	26.4	28.3	28.2	29.8	30.6	31.4	31.5	33.3	33.7	35.2	36.7	< 0.001
Preexisting Diabetes	1.0	1.2	1.0	1.2	1.4	1.4	1.4	2.2	2.6	2.8	2.8	< 0.001
Preexisting Hypertension	4.1	4.7	4.9	4.9	5.7	6.2	5.8	4.7	4.9	6.3	6.1	< 0.001
Smoking	9.9	10.8	11.3	11.5	11.6	10.7	10.7	8.8	8.7	9.0	8.1	< 0.001
Drug/Alcohol Use	5.3	6.6	7.1	7.3	8.7	10.4	11.1	8.9	8.9	9.6	9.9	< 0.001
Mental Health Condition	7.7	9.2	9.7	10.1	12.5	16.9	20.4	16.6	17.6	20.0	20.8	< 0.001
Sickle Cell Anemia	0.5	0.4	0.5	0.6	0.6	0.6	0.5	0.4	0.5	0.7	0.5	0.3571
Anemia	18.4	20.1	20.2	22.3	23.3	19.4	21.3	15.9	17.3	21.6	24.1	0.016
Gestational Diabetes	6.5	7.1	7.6	7.7	8.8	8.5	8.6	4.3	4.7	6.4	7.2	< 0.001
Gestational Hypertension	8.5	8.2	8.8	9.4	9.5	10.0	11.2	8.1	9.3	11.2	12.9	< 0.001
Infection (non-STI, non-COVID-19)	14.9	20.8	20.8	21.6	23.7	25.6	21.2	16.6	17.1	17.1	17.2	< 0.001
Sexually Transmitted Infection	0.4	0.6	0.6	0.7	1.3	3.9	3.7	2.9	3.3	4.6	4.7	< 0.001
Asthma	11.4	12.9	13.0	13.4	14.6	14.9	16.0	13.4	13.6	13.8	14.4	< 0.001
Sleep Disorder	0.2	0.3	0.4	0.3	0.5	0.5	0.6	0.6	0.4	0.5	0.6	< 0.001
Autoimmune Disorder	0.4	0.5	0.4	0.4	0.5	0.4	0.4	0.5	0.5	0.4	0.5	0.593
Malignancy	0.1	0.1	0.1	0.1	0.1	0.1	0.1	0.1	0.1	0.1	0.1	0.824
Dispilidemia	0.1	0.2	0.3	0.3	0.4	0.3	0.5	0.5	0.5	0.4	0.5	< 0.001
Previous Cesarean Section	18.8	20.0	20.0	20.9	21.3	22.3	21.9	21.0	21.7	23.2	21.7	< 0.001
Previous PTB	1.5	1.8	2.2	2.3	2.2	2.3	2.3	1.8	1.8	2.4	2.2	0.002
IPI < 18 months	18.5	18.6	18.7	18.9	19.0	18.7	18.6	18.9	18.9	18.4	16.8	0.062
IPI > 59 months	13.0	13.9	13.7	14.4	15.1	16.5	16.6	19.2	20.1	20.4	17.1	< 0.001
< 3 of prenatal care visits	2.8	2.8	2.9	3.0	3.1	3.6	4.0	3.9	3.8	4.1	3.9	< 0.001
Housing insecurity	0.3	0.6	0.6	0.7	0.9	1.1	1.2	1.3	1.5	1.6	1.4	< 0.001

© 2024 Jelliffe-Pawlowski LL et al. *JAMA Network Open.*

eTable 5. (continued)

	Non-Public Insurance											
	2011	2012	2013	2014	2015	2016	2017	2018	2019	2020	2021	Trend
**Black** (Continued)												*P* =
**Protective Factors** (RR < 1.00, *P* < .05)												
WIC participation	48.4	50.5	49.5	47.6	45.4	41.8	40.5	36.1	34.3	31.8	28.4	< 0.001
Mom born outside the US	13.5	13.5	14.1	15.0	15.4	15.2	15.5	16.4	14.5	14.0	15.3	< 0.001
**Risk Factors** (RR > 1.00, P < .05)												
Age > 34 years	19.9	20.7	21.1	22.2	22.9	23.8	24.6	26.7	25.1	27.9	29.1	< 0.001
< 12 years education	7.0	6.5	5.6	5.1	4.6	4.3	3.8	3.6	3.5	2.8	2.4	< 0.001
FIPS 2	16.3	15.9	15.2	14.2	15.7	15.9	16.2	15.3	15.4	16.8	16.0	0.311
Pre-pregnancy BMI < 18.5 kg/m^2	3.1	3.2	2.4	2.8	2.9	2.5	2.6	2.2	2.7	2.1	2.1	< 0.001
Pre-pregnancy BMI ≥30 kg/m^2	27.9	28.2	28.0	30.6	30.9	30.9	33.6	34.8	35.9	37.5	37.6	< 0.001
Preexisting Diabetes	1.1	0.9	0.9	1.2	1.2	1.3	1.5	2.2	2.1	2.5	2.6	< 0.001
Preexisting Hypertension	4.6	5.1	4.9	5.2	5.8	6.3	6.4	5.1	5.8	6.6	6.9	< 0.001
Smoking	4.2	4.8	4.0	4.0	3.9	3.5	3.1	2.7	2.6	2.6	2.1	< 0.001
Drug/Alcohol Use	2.8	3.3	3.3	3.3	3.9	4.1	4.5	3.1	3.0	3.6	2.8	0.607
Mental Health Condition	5.7	6.7	7.6	7.7	9.7	13.7	16.4	12.5	13.1	15.7	16.6	< 0.001
Sickle Cell Anemia	0.4	0.5	0.4	0.5	0.5	0.5	0.5	0.4	0.4	0.4	0.3	0.131
Anemia	22.0	24.3	25.7	28.0	29.2	27.0	29.9	21.0	22.8	25.1	27.3	0.015
Gestational Diabetes	8.0	8.6	9.6	9.1	9.5	8.9	9.0	5.2	5.9	7.3	8.0	< 0.001
Gestational Hypertension	7.7	8.5	9.3	9.6	10.7	11.1	12.8	9.9	11.7	14.2	15.7	< 0.001
Infection (non-STI, non-COVID-19)	11.2	14.7	14.8	14.3	16.9	19.3	13.7	8.3	8.3	8.8	8.9	< 0.001
Asthma	9.5	11.1	11.0	11.7	12.4	13.0	14.7	11.2	11.5	12.0	12.1	< 0.001
Sleep Disorder	0.2	0.5	0.6	0.5	0.6	0.8	0.9	0.7	0.6	1.0	1.0	< 0.001
Autoimmune Disorder	0.4	0.6	0.6	0.7	0.7	0.8	0.5	0.6	0.4	0.5	0.7	0.518
Malignancy	0.1	0.1	0.1	0.1	0.1	0.1	0.1	0.1	0.1	0.1	0.0	0.189
Dispilidemia	0.5	0.7	1.0	1.1	1.2	1.2	1.5	1.2	1.1	1.2	1.1	< 0.001
Previous Cesarean Section	16.4	16.3	17.8	18.2	18.1	18.8	19.3	18.3	19.2	19.1	18.7	< 0.001
Previous PTB	1.3	1.2	1.6	1.8	0.5	1.7	1.8	1.2	1.7	1.9	2.0	0.002
IPI > 59 months	14.4	14.7	15.7	15.5	15.7	15.4	15.7	18.0	18.2	19.0	15.0	< 0.001
< 3 of prenatal care visits	1.3	1.3	0.1	1.2	1.4	1.2	1.3	0.9	1.0	1.5	1.1	0.251
Housing insecurity	0.2	0.2	0.3	0.3	0.2	0.3	0.3	0.3	0.3	0.3	0.3	0.038
	Public Insurance											
**Hawaiian/ Pacific Islander**												
**Protective Factors** (RR < 1.00, *P* < .05)												
Public insurance for prenatal care	96.2	97.2	96.3	96.0	95.6	96.8	95.9	96.5	95.7	95.8	95.5	0.232
WIC participation	77.9	77.0	75.8	72.7	70.6	68.9	65.4	63.1	57.6	52.5	53.1	< 0.001
**Risk Factors** (RR > 1.00, P < .05)												
Age > 34 years	11.6	11.4	11.6	12.0	10.4	13.2	16.3	15.5	14.6	14.6	16.1	< 0.001
Nulliparous	29.8	31.3	28.1	26.6	24.4	29.5	26.4	27.3	31.2	34.6	28.6	0.613
Preexisting Diabetes	1.4	1.1	1.7	2.3	2.2	1.9	2.0	3.5	4.3	4.9	4.9	< 0.001
Preexisting Hypertension	3.2	3.5	3.3	3.2	3.9	3.5	4.0	3.3	3.1	4.4	5.7	0.061
Smoking	7.3	10.2	10.7	9.6	9.7	7.6	8.4	7.8	6.1	6.5	7.3	0.005
Drug/Alcohol Use	2.5	1.6	3.2	2.8	2.9	4.6	5.1	3.0	2.4	4.4	3.7	0.006

© 2024 Jelliffe-Pawlowski LL et al. *JAMA Network Open.*

eTable 5. (continued)

	Public Insurance (Continued)											
	**2011**	**2012**	**2013**	**2014**	**2015**	**2016**	**2017**	**2018**	**2019**	**2020**	**2021**	**Trend**
**Hawaiian/ Pacific Islander** (Continued)												*P* =
**Risk Factors** (RR > 1.00, P < .05) (Continued)												
Mental Health Condition	3.6	3.2	4.9	4.4	6.5	11.0	13.2	9.3	7.9	12.6	11.6	< 0.001
Anemia	12.9	15.1	13.1	14.3	18.0	13.2	13.2	13.6	10.5	14.5	17.5	0.710
Gestational Diabetes	13.1	13.7	15.6	16.5	13.6	15.4	19.2	6.9	9.8	11.6	13.1	0.015
Gestational Hypertension	7.2	7.3	8.4	10.3	6.0	10.8	12.3	9.9	7.8	12.2	10.2	< 0.001
Infection (non-STI, non-COVID-19)	10.8	15.9	14.0	18.1	18.8	24.0	15.5	13.9	13.9	15.4	17.8	0.027
Autoimmune Disorder	0.2	0.2	0.1	1.0	0.4	0.1	0.1	0.2	0.2	0.8	0.4	0.610
Dispilidemia	0.1	0.3	0.1	0.2	0.5	0.4	0.8	0.2	0.9	0.6	0.6	0.012
Previoous Cesarean Section	17.5	17.6	19.8	19.9	20.8	19.1	21.2	21.4	20.2	18.6	19.0	0.142
Previous PTB	0.5	1.0	1.7	2.0	2.0	1.5	1.3	1.5	1.3	2.1	1.2	0.163
IPI < 18 months	30.0	29.6	28.7	28.7	29.3	27.8	32.0	31.8	28.9	21.9	23.7	0.016
IPI > 59 months	10.0	8.4	11.0	12.3	10.8	11.2	11.5	13.4	13.9	11.8	10.4	0.014
< 3 of prenatal care visits	4.1	3.2	4.1	4.2	3.3	3.7	5.5	4.8	6.0	4.8	5.7	0.017
Housing Insecurity	0.2	0.2	0.1	0.3	0.7	0.5	0.9	0.6	0.2	0.6	0.2	0.164
	**Non-Public Insurance**											
**Risk Factors** (RR > 1.00, P < .05)												
Age > 34 years	19.9	21.6	21.1	10.0	22.7	21.2	26.5	23.5	27.2	28.0	27.3	< 0.001
Nulliparous	36.9	35.7	36.9	34.8	36.7	33.5	31.5	36.3	36.3	35.3	36.3	0.523
Preexisting Diabetes	1.7	1.9	1.7	1.3	2.3	2.3	3.0	4.9	6.2	4.2	4.2	< 0.001
Preexisting Hypertension	4.5	3.9	3.0	4.7	4.3	6.7	5.6	3.4	4.2	6.6	6.9	0.003
Drug/Alcohol Use	0.6	1.0	0.7	1.7	1.1	1.9	1.9	1.0	2.4	1.8	1.9	0.005
Mental Health Condition	2.4	3.1	2.5	4.5	4.3	9.9	12.6	7.5	10.5	13.2	14.8	< 0.001
Gestational Diabetes	15.2	17.7	18.8	17.8	17.3	17.2	16.4	8.8	10.1	13.5	14.8	< 0.001
Gestational Hypertension	5.6	8.7	8.8	8.2	10.8	8.7	12.6	9.2	9.1	13.0	13.4	< 0.001
Infection (non-STI, non-COVID-19)	8.2	8.3	11.2	10.7	14.9	19.9	12.5	7.6	6.2	8.1	8.7	0.331
Sleep Disorder	0.2	0.8	0.7	0.4	0.6	0.1	0.6	0.7	0.6	0.7	0.5	0.723
Autoimmune Disorder	0.2	0.5	0.4	0.5	0.2	0.6	0.3	0.7	0.5	0.8	0.3	0.287
Previous PTB	1.2	0.7	1.9	1.5	0.8	1.7	1.7	0.8	1.8	2.7	2.6	0.005
IPI > 59 months	13.1	11.9	11.7	13.2	12.6	12.9	13.9	16.1	17.3	14.9	14.8	0.001
< 3 of prenatal care visits	1.7	1.3	1.8	1.2	1.3	1.7	2.1	2.4	1.6	2.4	1.5	0.243
	**Public Insurance**											
**Hispanic**												
**Protective Factors** (RR < 1.00, *P* < .05)												
Public insurance for prenatal care	98.5	98.0	98.0	97.1	96.9	96.8	96.5	97.2	97.1	97.1	97.0	< 0.001
WIC participation	92.0	92.1	91.4	90.6	89.2	86.7	94.4	83.0	79.5	75.1	73.5	< 0.001
Nulliparous	31.7	31.7	31.3	30.6	29.9	29.2	29.2	29.7	30.1	30.0	30.3	< 0.001
FIPS 2	10.0	9.9	10.7	10.9	10.4	10.9	10.7	10.9	10.3	10.1	10.3	< 0.001
FIPS 3	24.3	24.4	25.1	25.7	25.6	26.0	26.4	26.4	26.3	26.5	27.6	< 0.001
FIPS 4	4.4	4.4	4.4	4.7	4.9	4.9	5.4	6.1	6.4	6.5	6.8	< 0.001
FIPS 5-6	1.0	1.0	1.0	1.0	1.1	1.1	1.1	1.2	1.3	1.3	1.3	< 0.001

© 2024 Jelliffe-Pawlowski LL et al. *JAMA Network Open.*

	**Public Insurance** (Continued)											
	**2011**	**2012**	**2013**	**2014**	**2015**	**2016**	**2017**	**2018**	**2019**	**2020**	**2021**	**Trend**
**Hispanic** (Continued)												*P* =
**Risk Factors** (RR > 1.00, P < .05)												
Age < 18 years	4.6	4.2	3.6	3.2	2.9	2.6	2.4	2.2	2.0	2.0	1.8	< 0.001
Age > 34 years	12.1	12.5	13.0	13.4	14.0	14.8	15.2	15.4	16.1	16.3	16.3	< 0.001
< 12 years education	48.0	44.8	41.9	39.9	37.5	35.3	33.3	33.0	31.4	30.0	27.3	< 0.001
Mom born outside the US	58.6	56.4	53.4	52.3	51.4	50.2	48.5	47.8	46.7	45.0	43.3	< 0.001
Pre-pregnancy BMI < 18.5 kg/m^2	2.7	2.6	2.6	2.5	2.5	2.5	2.3	2.2	2.0	1.9	1.8	< 0.001
Pre-pregnancy BMI ≥ 30 kg/m^2	25.3	26.4	27.7	28.4	29.1	30.1	31.8	33.3	34.5	35.8	36.6	< 0.001
Preexisting Diabetes	1.0	1.0	1.0	1.0	1.1	1.2	1.4	2.3	2.3	2.6	3.0	< 0.001
Preexisting Hypertension	1.3	1.5	1.6	1.7	1.9	2.2	2.2	1.7	1.9	2.3	2.6	< 0.001
Smoking	1.4	1.6	1.5	1.6	1.8	1.9	1.8	1.5	1.5	1.6	1.6	0.002
Drug/Alcohol Use	1.1	1.4	1.6	1.8	2.1	2.6	2.8	2.2	2.2	2.5	2.6	< 0.001
Mental Health Condition	2.1	2.5	2.9	3.3	3.9	5.1	6.1	5.0	5.5	6.8	7.6	< 0.001
Sickle Cell Anemia	0.0	0.0	0.0	0.0	0.0	0.0	0.0	0.0	0.0	0.0	0.0	0.630
Anemia	10.5	11.4	11.9	13.7	13.3	10.6	11.1	7.8	8.2	10.4	12.8	< 0.001
Gestational Diabetes	9.2	10.2	10.4	10.9	11.1	11.9	12.3	6.9	6.7	8.6	9.9	< 0.001
Gestational Hypertension	5.6	5.8	6.1	6.3	6.7	7.1	8.1	5.4	5.8	7.5	9.0	< 0.001
Infection (non-STI, non-COVID-19)	6.5	9.2	10.0	10.7	12.5	15.7	13.4	10.3	9.5	11.7	12.0	< 0.001
Sexually Transmitted Infection	0.1	0.1	0.2	0.3	0.6	1.2	1.4	1.0	1.1	1.4	1.6	< 0.001
Asthma	2.9	3.2	3.4	3.7	4.0	4.2	4.7	3.8	3.8	4.2	4.3	< 0.001
Sleep Disorder	0.1	0.1	0.1	0.1	0.2	0.1	0.2	0.1	0.2	0.2	0.2	< 0.001
Autoimmune Disorder	0.2	0.2	0.2	0.2	0.3	0.2	0.2	0.2	0.2	0.2	0.3	0.330
Malignancy	0.0	0.0	0.0	0.1	0.1	0.1	0.1	0.1	0.0	0.1	0.1	< 0.001
Dispilidemia	0.1	0.1	0.2	0.2	0.2	0.3	0.3	0.3	0.3	0.3	0.4	< 0.001
Previous Cesarean Section	18.4	18.7	18.9	19.3	19.7	20.0	20.0	19.7	19.7	19.7	19.0	< 0.001
Previous PTB	0.6	0.8	1.0	1.1	1.1	1.1	1.2	0.8	1.0	1.4	1.5	< 0.001
IPI < 18 months	16.3	16.0	16.0	15.9	16.2	16.4	16.4	16.4	15.5	15.4	15.2	< 0.001
IPI > 59 months	16.6	17.5	18.2	19.2	20.1	20.6	20.7	23.2	23.3	23.7	20.2	< 0.001
< 3 of prenatal care visits	1.0	1.1	1.2	1.3	1.4	1.6	1.7	1.8	1.7	1.8	1.9	< 0.001
Housing Insecurity	0.1	0.1	0.1	0.1	0.2	0.2	0.2	0.2	0.2	0.2	0.2	< 0.001
Intimate Partner Volence	0.0	0.0	0.0	0.0	0.0	0.1	0.1	0.0	0.1	0.1	0.1	< 0.001
**Protective Factors** (RR < 1.00, *P* < .05)												
FIPS 4	4.8	4.6	4.6	4.6	4.8	4.6	4.0	3.9	3.8	3.9	4.2	< 0.001
FIPS 5-6	0.6	0.6	0.6	0.6	0.7	0.7	0.7	0.7	0.9	0.9	0.9	< 0.001
> 12 years education	61.5	55.5	56.9	58.9	60.7	61.4	62.9	63.9	64.2	63.5	64.1	< 0.001
	**Non-Public Insurance**											
**Risk Factors** (RR > 1.00, P < .05)												
Age < 18 years	2.3	2.1	1.7	1.3	0.2	1.0	0.8	0.7	0.7	0.6	0.5	< 0.001
Age > 34 years	18.5	18.6	19.1	19.8	20.3	20.8	21.8	22.3	21.8	22.0	22.4	< 0.001
< 12 years education	14.0	13.0	11.4	10.2	9.1	8.4	7.5	6.8	6.6	6.0	5.1	< 0.001
Mom born outside the US	33.7	32.1	30.7	29.9	29.5	29.0	27.6	26.6	26.1	24.9	23.5	< 0.001

© 2024 Jelliffe-Pawlowski LL et al. *JAMA Network Open.*

eTable 5. (continued)

	Non-Public Insurance (Continued)											
	**2011**	**2012**	**2013**	**2014**	**2015**	**2016**	**2017**	**2018**	**2019**	**2020**	**2021**	**Trend**
**Hispanic** (Continued)												*P* =
**Risk Factors** (RR > 1.00, P < .05) (Continued)												
Pre-pregnancy BMI < 18.5 kg/m^2	2.1	2.2	2.1	2.1	2.1	1.9	1.8	1.7	1.6	1.5	1.4	< 0.001
Pre-pregnancy BMI ≥ 30 kg/m^2	24.5	25.1	25.6	26.3	27.3	28.6	30.7	32.4	33.4	35.2	35.3	< 0.001
Preexisting Diabetes	0.9	0.9	1.0	1.0	1.1	1.5	1.4	2.4	2.3	2.6	2.4	< 0.001
Preexisting Hypertension	1.9	1.9	2.1	2.1	2.4	2.8	2.8	2.3	2.4	2.9	3.4	< 0.001
Smoking	1.0	1.1	1.1	1.0	1.0	0.9	0.9	0.7	0.7	0.6	0.6	< 0.001
Drug/Alcohol Use	0.8	1.0	1.1	1.1	1.1	1.4	1.4	1.1	1.0	1.1	1.0	< 0.001
Mental Health Condition	2.9	3.4	4.0	4.6	5.5	8.0	10.0	8.4	8.5	10.2	11.8	< 0.001
Anemia	12.4	13.1	14.1	16.4	16.4	14.5	15.7	12.1	12.2	13.8	16.1	< 0.001
Gestational Diabetes	10.2	11.0	11.1	11.4	11.4	11.2	11.7	7.8	8.1	9.7	11.1	< 0.001
Gestational Hypertension	5.9	5.7	6.3	6.5	7.1	7.4	8.6	6.9	7.9	9.6	10.9	< 0.001
Infection (non-STI, non-COVID-19)	5.8	7.7	7.9	8.3	10.5	14.9	10.3	6.2	6.2	7.6	8.2	< 0.001
Sexually Transmitted Infection	0.1	0.1	0.1	0.1	0.4	1.4	1.6	1.3	1.4	1.6	1.8	< 0.001
Asthma	4.3	4.9	5.2	5.4	5.9	6.4	6.8	5.7	5.9	6.6	6.8	< 0.001
Sleep Disorder	0.2	0.2	0.3	0.3	0.3	0.4	0.4	0.4	0.3	0.4	0.5	< 0.001
Autoimmune Disorder	0.4	0.4	0.4	0.5	0.5	0.4	0.5	0.4	0.4	0.4	0.5	0.302
Malignancy	0.0	0.1	0.1	0.1	0.1	0.1	0.1	0.1	0.1	0.1	0.1	0.001
Dispilidemia	0.6	0.8	0.9	1.0	1.2	1.3	1.5	1.3	1.1	1.0	1.1	< 0.001
Previous Cesarean Section	16.2	16.4	16.4	16.9	17.0	17.4	17.6	16.7	17.1	16.5	15.9	0.174
Previous PTB	0.8	1.0	1.1	1.1	1.1	1.2	1.2	0.9	1.1	1.3	1.5	< 0.001
IPI < 18 months	14.0	14.0	13.8	13.7	13.8	14.0	13.8	13.9	13.7	13.4	13.3	< 0.001
IPI > 59 months	16.0	15.7	16.0	16.7	16.4	16.6	16.4	18.4	18.2	18.0	14.5	< 0.001
< 3 of prenatal care visits	0.9	0.9	1.0	1.0	1.0	0.9	1.0	0.9	1.0	0.9	0.8	0.627
Housing Insecurity	0.0	0.0	0.0	0.0	0.1	0.1	0.1	0.1	0.1	0.1	0.1	< 0.001
Intimate Partner Volence	0.0	0.0	0.0	0.0	0.0	0.1	0.0	0.1	0.1	0.0	0.0	< 0.001
	**Public Insurance**											
**White**												
**Protective Factors** (RR < 1.00, *P* < .05)												
> 12 years education	44.3	45.7	16.6	47.5	48.7	49.7	51.1	50.4	49.9	48.0	48.1	< 0.001
Public insurance for prenatal care	96.8	96.4	96.0	95.1	95.3	95.2	95.0	95.6	94.9	94.5	94.5	< 0.001
WIC participation	73.0	92.7	71.9	70.7	66.9	63.1	60.3	59.5	56.5	55.7	53.2	< 0.001
Nulliparous	40.0	39.3	38.3	37.3	36.5	35.0	33.9	34.1	33.8	34.0	33.4	< 0.001
Mom born outside the US	14.4	15.0	15.5	17.1	17.6	19.2	20.7	20.7	20.9	22.2	22.4	< 0.001
**Risk Factors** (RR > 1.00, P < .05)												
Age < 18 years	2.1	2.0	1.6	1.3	1.1	1.0	0.9	0.9	1.0	0.9	0.9	< 0.001
Age > 34 years	9.9	10.5	10.9	11.9	12.3	13.8	14.5	15.9	16.7	17.2	18.4	< 0.001
< 12 years education	16.0	15.2	13.7	13.2	12.1	11.4	10.8	11.7	11.8	11.2	10.4	< 0.001
FIPS 3	24.9	25.3	25.5	25.7	25.5	25.8	25.2	25.1	25.4	32.3	32.3	< 0.001
Pre-pregnancy BMI < 18.5 kg/m^2	5.1	5.1	5.0	5.0	5.1	4.9	4.9	4.7	4.3	4.0	4.1	< 0.001
Pre-pregnancy BMI ≥ 30 kg/m^2	21.8	22.8	23.3	23.6	23.3	24.0	24.6	25.5	26.9	28.3	28.6	< 0.001
Preexisting Diabetes	0.7	0.8	0.7	0.8	0.8	1.0	0.9	1.5	1.7	1.8	1.7	< 0.001

© 2024 Jelliffe-Pawlowski LL et al. *JAMA Network Open.*

eTable 5. (continued)

	**Public Insurance** (Continued)											
	**2011**	**2012**	**2013**	**2014**	**2015**	**2016**	**2017**	**2018**	**2019**	**2020**	**2021**	**Trend**
**White** (Continued)												*P* =
**Risk Factors** (RR > 1.00, P < .05) (Continued)												
Preexisting Hypertension	2.0	2.1	2.2	2.4	2.6	3.1	3.0	2.3	2.6	3.0	3.2	< 0.001
Smoking	17.4	17.9	17.5	17.2	17.0	15.9	14.7	13.4	12.5	11.4	10.1	< 0.001
Drug/Alcohol Use	5.9	6.7	7.1	7.4	8.1	9.8	10.2	8.0	7.5	8.0	8.0	< 0.001
Mental Health Condition	9.3	10.5	11.1	11.7	14.2	20.6	22.6	17.6	18.5	19.5	20.4	< 0.001
Anemia	9.6	10.8	11.1	11.9	12.2	9.9	10.3	8.2	8.5	9.8	12.1	< 0.001
Gestational Diabetes	6.9	8.1	8.5	8.6	8.7	8.9	9.3	6.1	5.9	7.3	8.1	< 0.001
Gestational Hypertension	5.9	6.5	6.3	6.7	7.0	7.0	8.2	6.2	6.6	8.3	9.1	< 0.001
Infection (non-STI, non-COVID-19)	10.3	14.1	14.1	14.7	16.2	18.7	14.7	11.2	10.3	10.8	12.0	< 0.001
Sexually Transmitted Infection	0.1	0.2	0.2	0.3	0.7	2.2	2.2	1.7	2.0	2.3	2.5	< 0.001
Asthma	7.2	8.3	8.3	8.7	9.1	9.4	9.4	7.8	7.8	8.0	7.4	0.592
Sleep Disorder	0.2	0.3	0.4	0.3	0.4	0.4	0.4	0.4	0.4	0.4	0.5	< 0.001
Autoimmune Disorder	0.4	0.5	0.4	0.5	0.5	0.6	0.6	0.5	0.5	0.5	0.6	0.003
Malignancy	0.1	0.1	0.1	0.1	0.1	0.1	0.1	0.1	0.1	0.1	0.1	0.594
Dispilidemia	0.1	0.2	0.2	0.2	0.3	0.4	0.4	0.3	0.2	0.4	0.5	< 0.001
Previous Cesarean Section	15.3	16.3	16.8	17.1	17.6	18.1	18.4	18.4	18.8	17.5	18.4	< 0.001
Previous PTB	1.1	1.3	1.4	1.5	0.1	1.3	1.4	1.2	1.3	1.4	1.6	0.005
IPI < 18 months	17.3	17.5	17.3	17.7	18.2	18.5	18.7	18.9	18.8	17.9	16.9	0.001
IPI > 59 months	11.4	11.5	12.4	13.0	13.3	13.9	13.8	16.4	16.5	17.0	13.6	< 0.001
< 3 of prenatal care visits	2.0	2.1	2.3	2.4	2.6	2.7	3.1	3.2	3.1	3.3	3.3	< 0.001
Housing Insecurity	0.3	0.5	0.6	0.6	0.7	0.8	0.9	0.8	0.8	0.9	0.8	< 0.001
Intimate Partner Volence	0.1	0.1	0.1	0.1	0.1	0.1	0.1	0.1	0.1	0.1	0.1	0.019
	**Non-Public Insurance**											
**Protective Factors** (RR < 1.00, *P* < .05)												
> 12 years education	82.9	83.5	84.1	84.9	85.3	86.1	87.0	87.1	86.5	86.2	86.4	< 0.001
Mom born outside the US	12.8	13.0	13.1	13.3	13.9	13.8	14.5	14.7	14.5	14.0	13.8	< 0.001
**Risk Factors** (RR > 1.00, P < .05)												
Age < 18 years	0.4	0.3	0.2	0.2	0.2	0.1	0.1	0.1	0.1	0.1	0.1	< 0.001
Age > 34 years	26.0	26.1	26.2	27.0	27.9	28.5	30.2	31.6	32.3	33.2	34.6	< 0.001
< 12 years education	1.7	1.5	1.3	1.2	1.0	1.0	0.9	0.9	0.8	0.8	0.7	< 0.001
FIPS 3	18.5	18.0	17.8	18.0	17.4	17.4	17.1	17.2	18.1	18.5	18.8	0.321
Pre-pregnancy BMI < 18.5 kg/m^2	3.6	3.8	3.6	3.6	3.6	3.3	3.4	3.3	3.1	2.9	2.8	< 0.001
Pre-pregnancy BMI ≥30 kg/m^2	14.5	14.3	14.6	14.9	15.1	15.8	16.4	17.0	17.7	18.4	19.0	< 0.001
Preexisting Diabetes	0.5	0.5	0.5	0.5	0.6	0.7	0.9	1.2	1.5	1.4	1.4	< 0.001
Preexisting Hypertension	1.8	1.8	1.8	1.9	2.1	2.6	2.6	2.1	2.4	2.9	3.5	< 0.001
Smoking	2.6	2.7	2.5	2.3	2.2	2.1	1.8	1.4	1.4	1.3	1.2	< 0.001
Drug/Alcohol Use	0.9	1.0	1.1	1.1	1.2	1.3	1.4	1.0	1.0	1.2	1.1	< 0.001
Mental Health Condition	4.6	5.1	5.7	6.3	7.5	10.8	13.1	11.2	12.2	15.1	16.8	< 0.001
Anemia	8.4	9.0	9.4	10.8	11.3	9.2	9.8	8.3	8.9	9.6	11.2	< 0.001
Gestational Diabetes	6.6	7.3	7.4	7.6	7.6	7.7	8.0	5.9	6.2	6.9	7.5	0.005
Gestational Hypertension	5.7	5.8	6.1	6.6	7.0	7.2	8.6	7.6	8.4	10.0	11.2	< 0.001

© 2024 Jelliffe-Pawlowski LL et al. *JAMA Network Open.*

eTable 5. (continued)

	Non-Public Insurance (Continued)											
	**2011**	**2012**	**2013**	**2014**	**2015**	**2016**	**2017**	**2018**	**2019**	**2020**	**2021**	**Trend** *P =*
**White** (Continued)												
**Risk Factors** (RR > 1.00, P < .05) (Continued)												
Infection (non-STI, non-COVID-19)	5.2	6.1	6.4	6.7	8.3	12.0	7.4	4.3	4.0	4.2	5.0	< 0.001
Asthma	4.7	4.9	5.3	5.8	6.5	6.8	7.2	6.1	6.4	7.1	7.3	< 0.001
Sleep Disorder	0.2	0.2	0.3	0.3	0.4	0.4	0.5	0.4	0.4	0.5	0.5	< 0.001
Autoimmune Disorder	0.6	0.7	0.7	0.8	0.9	0.8	0.9	0.8	0.9	0.9	0.9	< 0.001
Malignancy	0.1	0.1	0.1	0.1	0.1	0.1	0.1	0.1	0.1	0.1	0.1	0.390
Dispilidemia	0.3	0.5	0.5	0.6	0.6	0.6	0.7	0.7	0.8	0.8	0.8	< 0.001
Previous Cesarean Section	14.4	14.2	14.5	14.4	14.6	14.6	14.2	14.1	14.7	14.3	14.1	0.449
Previous PTB	0.9	1.0	1.1	1.1	1.0	1.1	1.0	1.0	1.1	1.2	1.4	< 0.001
IPI > 59 months	6.6	6.6	6.7	6.5	6.4	6.5	6.2	7.2	7.6	7.3	5.7	0.030
< 3 of prenatal care visits	0.4	0.4	0.4	0.3	0.3	0.3	0.4	0.3	0.3	0.4	0.3	0.021
Housing Insecurity	0.0	0.0	0.0	0.0	0.0	0.0	0.1	0.0	0.0	0.1	0.1	0.095
Public insurance for prenatal care	0.8	0.5	0.5	0.5	0.5	0.5	0.4	0.5	0.8	0.8	0.8	< 0.001
WIC participation	11.0	11.2	10.7	10.3	9.9	8.4	8.4	7.2	6.6	6.2	5.8	< 0.001
Nulliparous	45.8	46.1	45.9	46.2	45.7	45.4	45.5	45.8	45.7	45.7	46.2	0.311
	**Public Insurance**											
**Other**												
**Protective Factors** (RR < 1.00, *P* < .05)												
Public insurance for prenatal care	97.9	97.6	97.2	96.4	96.0	95.8	95.4	96.1	94.8	95.0	94.7	< 0.001
WIC participation	80.9	81.4	77.7	76.3	72.7	69.3	65.4	66.4	62.0	56.4	53.8	< 0.001
Nulliparous	41.0	40.1	39.6	38.5	36.1	35.3	33.6	33.8	33.9	33.4	33.3	< 0.001
Mom born outside the US	26.9	27.1	17.5	20.9	21.6	23.2	23.0	24.2	28.7	25.8	24.2	< 0.001
FIPS 5-6	4.9	3.7	4.8	3.8	4.0	3.6	3.9	3.9	3.0	3.3	3.3	< 0.001
**Risk Factors** (RR > 1.00, P < .05)												
Age > 34 years	10.4	10.7	10.7	11.1	11.7	13.6	13.8	14.1	15.9	15.9	17.3	< 0.001
Pre-pregnancy BMI < 18.5 kg/m^2	4.2	4.0	4.4	4.5	4.4	4.1	4.0	3.9	3.3	3.3	3.0	< 0.001
Pre-pregnancy BMI ≥ 30 kg/m^2	22.9	23.6	25.6	25.2	25.9	27.9	28.5	29.6	29.2	31.6	30.3	< 0.001
Preexisting Diabetes	0.8	1.3	1.1	0.6	1.0	1.3	1.2	1.7	2.2	2.5	2.4	< 0.001
Preexisting Hypertension	2.5	2.6	2.9	2.7	3.3	3.5	3.3	2.6	2.9	3.7	3.8	< 0.001
Smoking	11.5	11.2	12.9	12.1	12.1	12.0	11.4	10.2	8.4	7.7	7.6	< 0.001
Drug/Alcohol Use	5.1	6.2	7.4	7.6	8.9	9.8	9.6	7.9	6.6	7.9	8.7	< 0.001
Mental Health Condition	8.6	9.8	11.3	12.0	14.3	19.3	20.2	16.8	16.7	18.4	20.0	< 0.001
Anemia	12.1	13.9	14.2	16.0	17.4	14.6	14.1	12.1	12.8	14.7	17.0	0.014
Gestational Diabetes	9.5	11.2	9.4	10.4	10.2	11.4	10.3	6.6	7.0	7.8	9.2	< 0.001
Gestational Hypertension	7.4	7.8	7.2	8.7	8.3	8.5	9.7	7.8	8.1	8.9	11.4	< 0.001
Infection (non-STI, non-COVID-19)	11.2	14.4	15.0	16.9	17.6	19.8	14.7	11.9	10.6	13.7	13.3	< 0.001
Sexually Transmitted Infection	0.2	0.4	0.3	0.5	1.0	3.1	2.6	2.2	2.4	2.8	3.5	< 0.001
Sleep Disorder	0.2	0.3	0.4	0.2	0.5	0.5	0.6	0.3	0.5	0.5	0.7	< 0.001
Autoimmune Disorder	0.4	0.4	0.5	0.6	0.5	0.4	0.7	0.4	0.3	0.3	0.5	0.762
Malignancy	0.1	0.1	0.0	0.1	0.1	0.1	0.1	0.1	0.0	0.0	0.1	0.530
Dispilidemia	0.1	0.2	0.3	0.4	0.3	0.5	0.3	0.4	0.4	0.4	0.4	0.001

© 2024 Jelliffe-Pawlowski LL et al. *JAMA Network Open.*

	Public Insurance (Continued)											
	2011	2012	2013	2014	2015	2016	2017	2018	2019	2020	2021	Trend
Other (Continued)												P =
**Risk Factors** (RR > 1.00, P < .05) (Continued)												
Previous Cesarean Section	14.0	14.8	16.7	17.3	18.8	19.3	19.4	17.5	17.6	18.9	18.2	< 0.001
Previous PTB	1.1	1.1	1.4	1.6	1.4	1.7	1.5	1.3	1.8	1.7	2.1	< 0.001
IPI < 18 months	16.5	16.0	17.2	17.6	18.1	18.1	19.0	18.0	17.4	17.3	16.6	0.168
IPI > 59 months	12.7	13.2	13.2	14.7	15.5	14.8	16.3	18.3	18.7	19.1	16.9	< 0.001
< 3 of prenatal care visits	1.8	2.3	2.5	3.3	4.2	4.1	4.2	4.2	4.5	3.6	3.8	< 0.001
Housing Insecurity	0.3	0.7	0.7	0.7	1.1	1.0	1.1	0.9	0.8	1.2	1.0	< 0.001
	Non-Public Insurance											
**Protective Factors** (RR < 1.00, *P* < .05)												
> 12 years education	48.1	44.4	49.8	48.0	49.2	44.6	44.8	41.4	38.5	39.7	38.5	< 0.001
**Risk Factors** (RR > 1.00, P < .05)												
Age < 18 years	1.4	1.0	0.7	0.6	0.5	0.4	0.3	0.4	0.3	0.3	0.2	< 0.001
Age > 34 years	27.2	27.5	26.3	27.1	29.5	31.3	32.1	31.9	32.2	33.6	34.5	< 0.001
< 12 years education	2.7	2.0	1.6	1.3	1.2	1.1	0.8	0.8	0.7	0.8	0.5	< 0.001
FIPS 4	1.9	2.2	2.1	1.8	2.1	1.8	2.0	1.9	2.2	2.9	2.0	0.003
Pre-pregnancy BMI < 18.5 kg/m^2	3.8	4.1	3.9	4.0	3.4	3.4	3.6	3.4	3.5	3.1	3.0	< 0.001
Pre-pregnancy BMI ≥30 kg/m^2	17.0	15.5	17.2	15.8	17.7	18.0	18.9	20.0	20.0	21.3	21.5	< 0.001
Preexisting Diabetes	0.8	0.6	0.6	0.7	0.8	1.0	1.1	1.7	2.0	2.1	2.1	< 0.001
Preexisting Hypertension	2.7	2.6	2.3	2.3	2.5	3.3	3.3	2.5	2.9	3.5	4.1	< 0.001
Smoking	3.0	3.0	3.3	2.5	2.4	2.0	1.8	1.5	1.4	1.3	1.2	< 0.001
Drug/Alcohol Use	1.6	1.7	2.1	1.7	1.8	2.1	1.9	1.6	1.6	1.7	1.8	0.357
Mental Health Condition	5.5	6.0	6.7	6.9	8.1	11.4	13.1	12.1	13.3	14.7	17.0	< 0.001
Sickle Cell Anemia	0.1	0.1	0.1	0.0	0.1	0.0	0.0	0.0	0.0	0.0	0.0	< 0.001
Anemia	12.8	13.2	13.7	15.5	15.0	12.2	13.6	12.3	13.1	13.2	14.8	0.802
Gestational Diabetes	8.7	9.9	10.0	10.7	10.8	10.8	11.6	7.9	8.9	9.7	10.0	0,128
Gestational Hypertension	6.2	5.6	6.5	7.8	8.1	7.6	8.8	8.6	10.2	11.1	13.1	< 0.001
Infection (non-STI, non-COVID-19)	6.7	7.2	7.7	8.3	9.7	12.6	7.8	5.2	4.5	5.6	6.1	< 0.001
Sexually Transmitted Infection	0.1	0.2	0.2	0.1	0.7	2.2	3.0	2.1	2.1	2.4	2.9	< 0.001
Asthma	6.8	7.1	7.4	8.0	8.5	9.0	9.6	7.4	8.1	8.6	8.8	< 0.001
Sleep Disorder	0.3	0.4	0.4	0.3	0.5	0.5	0.6	0.5	0.6	0.7	0.6	< 0.001
Autoimmune Disorder	0.5	0.7	0.6	0.8	0.6	0.7	0.8	0.5	0.5	0.7	0.7	0.656
Malignancy	0.1	0.2	0.1	0.1	0.1	0.1	0.1	0.1	0.1	0.1	0.1	0.082
Dispilidemia	0.5	0.5	0.6	0.8	1.0	0.8	0.9	1.0	1.0	1.2	1.1	< 0.001
Previous Cesarean Section	13.8	13.2	13.9	14.6	14.7	15.3	15.6	14.1	13.9	14.3	14.1	0.154
Previous PTB	0.7	0.8	1.0	1.3	1.1	1.3	1.1	1.0	1.1	1.2	1.6	< 0.001
IPI < 18 months	14.3	14.0	14.3	13.9	14.2	14.6	14.5	14.4	2.0	13.7	13.1	< 0.001
IPI > 59 months	9.8	8.9	9.7	9.4	9.8	9.8	10.0	10.9	11.1	11.6	8.5	< 0.001
< 3 of prenatal care visits	0.6	0.5	0.8	0.6	0.7	0.6	0.6	0.5	0.8	0.8	0.7	0.060
Housing Insecurity	0.1	0.0	0.1	0.1	0.2	0.1	0.1	0.1	0.1	0.1	0.1	0.049
Intimate Partner Volence	0.0	0.1	0.1	0.1	0.0	0.1	0.1	0.1	0.1	0.0	0.1	0.373
Nulliparous	47.2	48.3	47.7	48.6	47.2	45.7	45.1	47.6	48.5	47.6	48.6	0.323

Abbreviations: BMI, body mass index; FIPS, Federal Information Processing Standard county code where 1 = most urban to 6 = most rural;[3] IPI, interpregnancy interval; PTB, preterm birth (gestational weeks < 37 completed weeks); STI, sexually transmitted infection; WIC, Nutritional program for Women, Infants, and Children[4]
Racial/ethnicity groups other than 'Hispanic" are non-Hispanic, 'Other' race/ethnicity = 'Indian (Asian)', 'Filipino', 'two or more races', 'other-specified', 'refused to state', and 'unknown'. Additional information about race/ethnicity groupings is included in eTable 1.

© 2024 Jelliffe-Pawlowski LL et al. *JAMA Network Open.*

eReferences

1. Centers for Disease Control and Prevention, National Center for Health Statistics. The International Classification of Diseases, Ninth Revision, Clinical Modification (ICD-9-CM). Accessed May 22, 2024. https://www.cdc.gov/nchs/icd/icd9cm.htm
2. Centers for Disease Control and Prevention, National Center for Health Statistics. The International Classification of Diseases, Tenth Revision, Clinical Modification (ICD-10-CM). Accessed May 22, 2024. https://www.cdc.gov/nchs/icd/icd-10-cm.htm
3. Centers for Disease Control and Prevention, National Center for Health Statistics. NCHS urban-rural classification scheme for counties. Accessed May 22, 2024. https://www.cdc.gov/nchs/data_access/urban_rural.htm
4. California Department of Public Health. California women, infants, & children program (WIC). Accessed May 22, 2024. https://www.cdph.ca.gov/Programs/CFH/DWICSN/Pages/Program-Landing1.aspx

© 2024 Jelliffe-Pawlowski LL et al. *JAMA Network Open.*
