# Supplementary material for: Risk and Protective Factors for Preterm Birth Among Racial, Ethnic, and Socioeconomic Groups in California
Source: JAMA Netw Open. 2024 Sep 27;7(9):e2435887. doi: 10.1001/jamanetworkopen.2024.35887 (PMC11437386; doi:10.1001/jamanetworkopen.2024.35887)
Supplement: Supplement 2. — Data Sharing Statement [file jamanetwopen-e2435887-s002.pdf]

Data Sharing Statement

Jelliffe-Pawlowski. Risk and Protective Factors for Preterm Birth Among Racial, Ethnic, and Socioeconomic Groups in California. *JAMA Netw Open*. Published October 02, 2024. doi:10.1001/jamanetworkopen.2024.35887

Data
Data available: No

Additional Information
Explanation for why data not available: This study leverages public health data available to researchers upon application from the California Department of Public Health. While the state of California does not allow the direct sharing of data with non-state-approved investigators, data is available, with approval from the Committee for the Protection of Human Subjects within the Health and Human Services Agency of the State of California, for vital records from the California Department of Public Health (CDPH) and for hospital discharge records from the California Department of Health Care Access and Information (HCAI).
